# The Architecture of Metabolism Maximizes Biosynthetic Diversity in the Largest Class of Fungi

**DOI:** 10.1093/molbev/msaa122

**Published:** 2020-05-18

**Authors:** Emile Gluck-Thaler, Sajeet Haridas, Manfred Binder, Igor V Grigoriev, Pedro W Crous, Joseph W Spatafora, Kathryn Bushley, Jason C Slot

**Affiliations:** m1 Department of Plant Pathology, The Ohio State University, Columbus, OH; m2 Department of Biological Sciences, University of Pittsburgh, Pittsburgh, PA; m3 US Department of Energy Joint Genome Institute, Lawrence Berkeley National Laboratory, Berkeley, CA; m4 TechBase, R-Tech GmbH, Regensburg, Germany; m5 Department of Plant and Microbial Biology, University of California, Berkeley, Berkeley, CA; m6 Westerdijk Fungal Biodiversity Institute, Utrecht, The Netherlands; m7 Department of Botany and Plant Pathology, Oregon State University, Corvallis, OR; m8 Department of Plant and Microbial Biology, University of Minnesota, Minneapolis, MN

**Keywords:** chemical ecology, fungi, metabolism, gene cluster

## Abstract

Ecological diversity in fungi is largely defined by metabolic traits, including the ability to produce secondary or “specialized” metabolites (SMs) that mediate interactions with other organisms. Fungal SM pathways are frequently encoded in biosynthetic gene clusters (BGCs), which facilitate the identification and characterization of metabolic pathways. Variation in BGC composition reflects the diversity of their SM products. Recent studies have documented surprising diversity of BGC repertoires among isolates of the same fungal species, yet little is known about how this population-level variation is inherited across macroevolutionary timescales. Here, we applied a novel linkage-based algorithm to reveal previously unexplored dimensions of diversity in BGC composition, distribution, and repertoire across 101 species of *Dothideomycetes*, which are considered the most phylogenetically diverse class of fungi and known to produce many SMs. We predicted both complementary and overlapping sets of clustered genes compared with existing methods and identified novel gene pairs that associate with known secondary metabolite genes. We found that variation among sets of BGCs in individual genomes is due to nonoverlapping BGC combinations and that several BGCs have biased ecological distributions, consistent with niche-specific selection. We observed that total BGC diversity scales linearly with increasing repertoire size, suggesting that secondary metabolites have little structural redundancy in individual fungi. We project that there is substantial unsampled BGC diversity across specific families of *Dothideomycetes*, which will provide a roadmap for future sampling efforts. Our approach and findings lend new insight into how BGC diversity is generated and maintained across an entire fungal taxonomic class.

## Introduction 

Plants, bacteria, and fungi produce the majority of the earth’s biochemical diversity. These organisms produce a remarkable variety of secondary/specialized metabolites (SMs) that can mediate ecological functions, including defense, resource acquisition, and mutualism. Standing SM diversity is often high at the population level, which may affect the rates of adaptation over microevolutionary timescales. For example, high intraspecific quantitative and qualitative chemotype diversity in plants can enable rapid adaptation to local biotic factors (Agrawal et al. 2012; Züst et al. 2012; Glassmire et al. 2016), and the ability to produce specific SMs is linked to ecological adaptation and host-specific interactions in fungi (Thynne et al. 2019; Kominek et al. 2019). However, the fate of population-level chemodiversity across longer timescales is not well explored in any of these lineages. We therefore sought to identify how metabolic variation is distributed across macroevolutionary timescales by profiling chemodiversity across a well-sampled taxonomic class.

The *Dothideomycetes*, which originated between 247 and 459 Ma (Beimforde et al. 2014), comprise the largest and arguably most phylogenetically diverse class of fungi. Currently, 19,000 species are recognized in 32 orders containing more than 1,300 genera (Zhang et al. 2011). *Dothideomycetes* are divided into two major subclasses, the Pleosporomycetidae (order Pleosporales) and Dothideomycetidae (orders Dothideales, Capnodiales, and Myriangiales), which correspond to the presence or absence, respectively, of pseudoparaphyses during development of the asci (Schoch et al. 2009). Several other orders await definitive placement.

*Dothideomycetes* also display a large diversity of fungal lifestyles and ecologies. The majority of *Dothideomycetes* are terrestrial and associate with phototrophic hosts as pathogens, saprobes, endophytes (Schoch et al. 2009), lichens (Nelsen et al. 2011), or ectomycorrhizal symbionts (Spatafora et al. 2012). Ancestral character state reconstructions predict that the ancestral ecology of *Dothideomycetes* was terrestrial and saprophytic, with multiple and independent transitions to other lifestyles (Schoch et al. 2006, 2009; Haridas et al. 2020). At least six orders have evolved plant pathogens, together affecting most crop species. The Pleosporales and Capnodiales represent two independent transitions to plant pathogens, mostly asexual, that cause significant economic losses and have been well sampled in previous genome sequencing efforts (Goodwin et al. 2011; Ohm et al. 2012; Oliver et al. 2012; Manning et al. 2013; Condon et al. 2018). Pleosporales consists primarily of nectrotrophs that rely on secondary metabolite (SM) toxins and suites of degradative enzymes for pathogenesis, whereas most Capnodiales are hemibiotrophs with fewer secondary metabolism, carbohydrate degradation, and proteolysis genes (Ohm et al. 2012). Stem-canker pathogens within Botrysphaeriales (*Neofusicoccum*, *Diplodia*, and *Botryosphearia*) (Phillips et al. 2013), fruit crop pathogens in Venturiales (*Venturia* spp.) (Gonzalez-Dominguez et al. 2017) and Myriangiales (*Elsinoë* spp.) (Liao and Chung 2008; Braga et al. 2019), represent additional transitions to plant pathogenesis. Several lichenized groups have arisen independently within the class, and clades of both freshwater and marine taxa have evolved multiple times within several orders (Schoch et al. 2009). Human and animal pathogens, including some taxa that can elicit allergies and asthma (Crameri et al. 2014), and rock-inhabiting fungi (Ruibal et al. 2009) are spottily distributed across the class.

This broad range of lifestyles is accompanied by extensive diversity of SMs, for which very few have known ecological roles (Muria‐Gonzalez et al. 2015). The *Dothideomycetes*, and several other ascomycete classes (Eurotiomycetes, Sordariomycetes, and Leotiomycetes), produce the greatest number and diversity of SMs across the fungal kingdom (Akimitsu et al. 2014; Spatafora and Bushley 2015). Economically important plant pathogens in the Pleosporales (*Alternaria*, *Bipolaris*, *Exserohilum*, *Leptosphaeria*, *Pyrenophora*, and *Stagonospora*), in particular, are known to produce host-selective toxins that confer the ability to cause disease in specific plant hosts (Walton and Panaccione 1993; Walton 1996; Wolpert et al. 2002; Ciuffetti et al. 2010; Pandelova et al. 2012; Akimitsu et al. 2014). Other toxins first identified in Pleosporales have roles in virulence but are not pathogenicity determinants, including depudecin (Wight et al. 2009) and solanapyrone (Kaur 1995). Although lichenized fungi are prolific SM producers (Oksanen 2006; Bertrand and Sorensen 2018; Calcott et al. 2018), lichenized *Dothideomycetes* genomes have not been sequenced here or elsewhere.

*Dothideomycetes* also produce some bioactive metabolites shared with more distantly related fungal classes. Sirodesmin, a virulence factor produced by *Leptosphaeria maculans*, for example, belongs to the same class of epipolythiodioxopiperazine (ETP) toxins as gliotoxin, an immunosuppressant produced by the eurotiomycete human pathogen *Aspergillus fumigatus* (Gardiner et al. 2005; Patron et al. 2007). Dothistromin, a polyketide metabolite produced by the pine pathogen *Dothistroma septosporum* shares ancestry with aflatoxin (Bradshaw et al. 2013), a mycotoxin produced by *Aspergillus* species that poses serious human health and environmental risks worldwide (Horn 2003; Wang and Tang 2004).

A majority of bioactive metabolites in *Dothideomycetes* are small-molecule SMs that are produced by biosynthetic gene clusters (BGCs) composed of enzymes, transporters, and regulators that contribute to a common SM pathway. Most of these BGCs are defined by four main classes of SM core signature enzymes: 1) nonribosomal peptide synthetases (NRPSs), 2) polyketide synthetases (PKSs), 3) terpene cyclases (TCs), and 4) dimethylallyl tryptophan synthases (DMATs) (Hoffmeister and Keller 2007). Fungal gene clusters are hotspots for genome evolution through gene duplication, loss, and horizontal transfer, which recombine pathways and generate diversity (Wisecaver et al. 2014). Additionally, recent studies have shown that gene clusters may evolve through recombination or shuffling of modular subunits of syntenic genes (Lind et al. 2017; Gluck-Thaler et al. 2018). Changes in BGC gene content often result in structural changes to the SM product(s), and therefore BGCs can be used to monitor the evolution of chemodiversity (Lind et al. 2017; Proctor et al. 2018). The most widely used methods for detecting BGCs rely on models of gene cluster composition based on putative functions in SM biosynthesis informed by a phylogenetically limited set of taxa, but gene function-agnostic methods are being developed (Slot and Gluck-Thaler 2019).

Here, we systematically assessed BGC richness and compositional diversity in the genomes of 101 *Dothideomycetes* species, most recently sequenced (Haridas et al. 2020). Using a newly benchmarked algorithm that identifies clustered genes of interest through the frequency of their co-occurrence with and around signature biosynthetic genes, we identified 3,399 putative BGCs, grouped into 719 unique BGC families, including five families of candidate 1,8-dihydroxynaphthalene (DHN) melanin clusters. The conservation of specific gene pairs across BGC types suggests that precise functional interactions contribute to the modular evolution of these loci. Numerous BGCs have either over- or underdispersed phylogenetic distributions, suggesting pathways have been differentially impacted by selection. In comparisons across species, BGC repertoire diversity increases linearly with repertoire size, reflecting a mode of metabolic evolution in these fungi that is likely distinct from that of plants. We found little overlap in BGC repertoires among genomes from different genera and project that a wealth of unique BGCs remain to be discovered within this fungal lineage.

## Results

### *Dothideomycetes* Contain Hundreds of BGC Families, a Small Fraction of Which Are Characterized

**Fig. 1.**
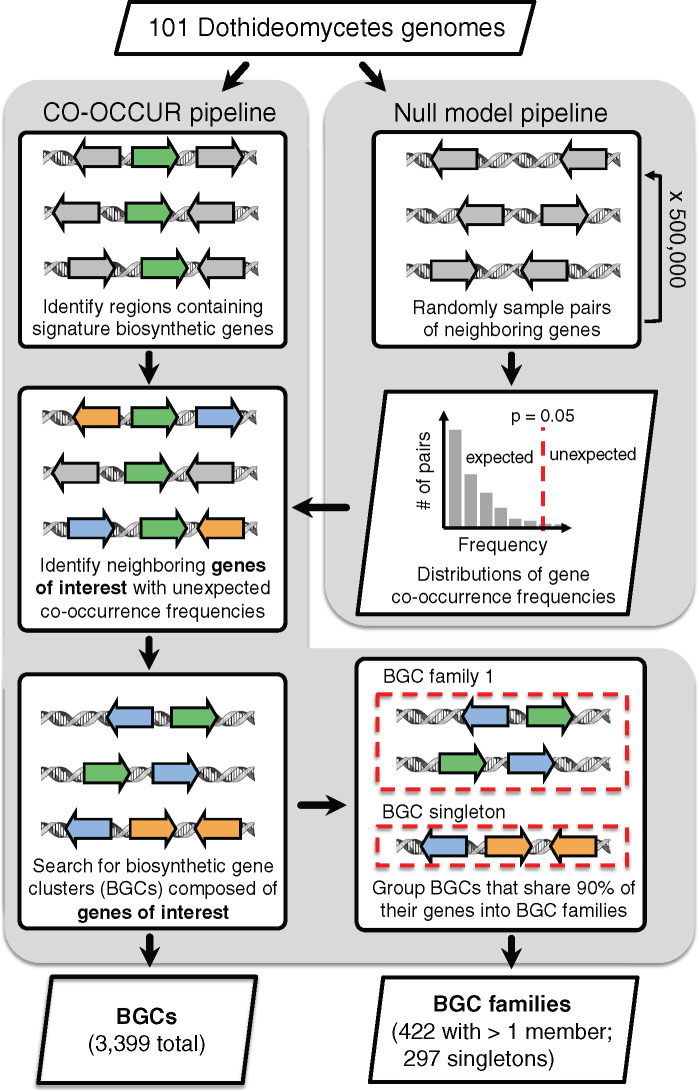
CO-OCCUR pipeline. The pipeline used genome annotations from 101 *Dothideomycetes*, and previously computed gene homolog groups consisting of both orthologs and paralogs (Haridas et al. 2020). BGCs were inferred by determining unexpectedly distributed shared gene homolog group pairs, determined according to a null distribution of randomly sampled gene pairs in the same genomes, and then a search for all clusters containing the gene homolog group pairs. The resulting BGCs were then consolidated into BGC families where members share ≥90% of gene homolog group content. A detailed pipeline is presented in supplementary figure SA, Supplementary Material online.

**Fig. 2.**
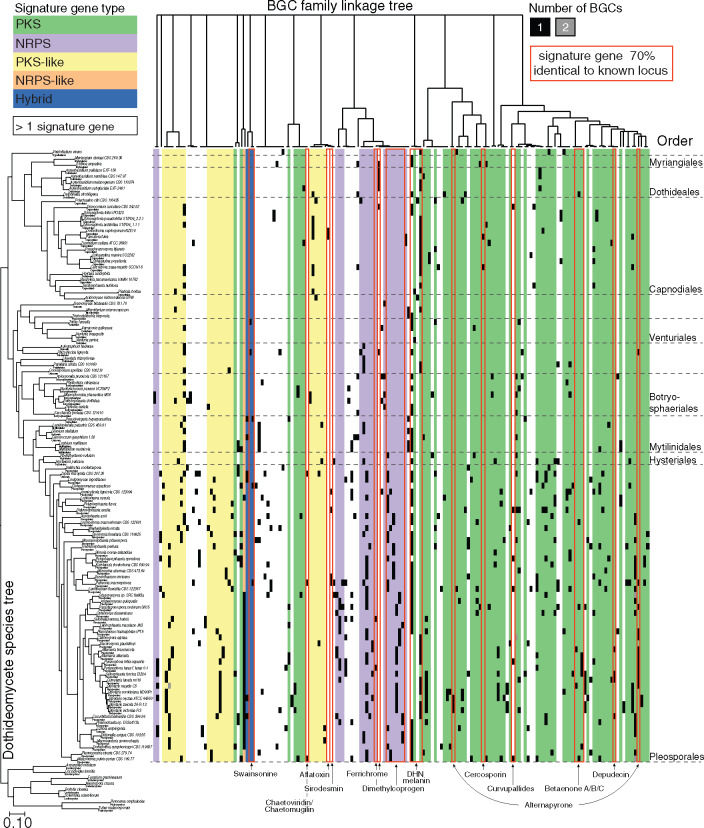
Diversity of the largest detected SM gene clusters across 101 *Dothideomycetes*. A maximum likelihood phylogenomic tree of 101 *Dothideomycetes* species (Haridas et al. 2020) corresponds to rows in a heatmap (right) that depicts the number of BGCs found in each genome, delimited by order (dotted line). Each cluster is assigned to a BGC family (column) defined by at least 90% similarity at the composition level. Only BGC families with ≥5 unique gene homolog groups per cluster are shown. A complete linkage tree (top) depicts relationships among BGC families, where distance is proportional to the Raup–Crick dissimilarity in BGC family composition. BGC families are colored according to their core signature biosynthetic genes, and BGC families with >1 signature gene are left uncolored. BGC families with signature genes ≥70% identical to characterized BGC signature genes in MIBiG are indicated by a labeled red box.

Using a novel cluster detection approach based on shared syntenic relationships among genes (CO-OCCUR, see Materials and Methods, fig. 1 and supplementary fig. SA, Supplementary Material online), we identified 332 gene homolog groups of interest (supplementary tables SA and SB, Supplementary Material online) whose members were organized into 3,399 candidate BGCs of at least two genes (supplementary table SC, Supplementary Material online) in 101 *Dothideomycetes* genomes (supplementary table SD, Supplementary Material online), representing an average of 33.7 BGCs per genome (SD = 15.4, supplementary fig. SB, Supplementary Material online). We grouped BGCs into 719 unique BGC families based on a minimum gene content similarity of 90%. Of these, 459 BGC families had four or more genes per BGC (fig. 2 and supplementary fig. SC, Supplementary Material online), and only nine of these BGC families were ever found more than once in any given genome (supplementary table SE, Supplementary Material online). According to standard practice, we classified BGC families based on the presence of biosynthetic signature genes: DMAT, PKS, PKS-like, NRPS, NRPS-like, HYBRID (containing both PKS and NRPS signature genes), and TC. We found that among all BGC families with greater than four genes, 186 contained only PKS and 29 contained only NRPS signature genes. Similarly, we detected 4 DMAT, 38 PKS-like, 16 NRPS-like, 3 HYBRID, and 3 TC-only BGC families. In total, 127 BGC families contained more than 1 type of signature gene, and 53 BGC families contained no signature gene at all but still consisted of genes found in significant co-occurrences. In total, 158 of CO-OCCUR BGCs corresponded to 32 unique MIBiG entries (supplementary table SG, Supplementary Material online) and 22 unique metabolites (supplementary table SF, Supplementary Material online).

Some CO-OCCUR detected BGCs matched MIBiG entries that encode nonhost-selective phytotoxins or other compounds with known roles in virulence to plants, particularly those in the large plant-pathogenic order Pleosporales. Several BGCs matching the dimethylcoprogen (extracellular siderophore) cluster, a virulence factor in the corn pathogen *Cochliobolus heterostrophus* (*Dothideomycetes*) and *Fusarium graminearum* (Sordariomycetes), were also found in most Pleosporales taxa (Oide et al. 2006). Two PKS BGCs encoding the nonhost-specific phytotoxin and DNA polymerase inhibitor, solanapyrone (Mizushina et al. 2002; Kasahara et al. 2010), and the related alternapyrone (Fujii et al. 2005), first identified in *Alternaria solani* (Mizushina et al. 2002; Fujii et al. 2005; Kasahara et al. 2010), were found across taxa primarily in the order Pleosporales, especially in the closely related Pleosporaceae, Leptosphaeriaceae, and Phaeosphaeriaceae families (fig. 2 and supplementary tables SF and SG, Supplementary Material online). In contrast, a BGC matching the sirodesmin (NRPS phytotoxin) cluster in *L. maculans* (Gardiner et al. 2004) and the depudecin (PKS histone deacetylase inhibitor) cluster in *Alternaria brassicicola* (Wight et al. 2009) were discontinuously distributed in a few unrelated species within Pleosporales (fig. 2 and supplementary tables SF and SG, Supplementary Material online). The only other putative host-selective toxin BGC matched the T-toxin (PKS) cluster from race T (C4) of *Cochliobolus heterostrophus* (only Race O (C5) included in this study), the fungus responsible for the devastating Southern Corn Leaf Blight (Daly 1982; Turgeon and Baker 2007). T-toxin cluster homologs were also detected in *Ampelomyces quisqualis* and *L. maculans* (supplementary table SG, Supplementary Material online).

Other CO-OCCUR BGCs matched MIBiG clusters from other ascomycete classes (Eurotiomycetes, Sordariomycetes), some of which have been previously detected in *Dothideomycetes*, whereas others were unexpected. The aflatoxin-like dothistromin clusters, which are fragmented into six miniclusters in *Dothistroma septorum* (BradshawSlot et al. 2013), were detected in *Dothistroma septorum* and the closely related *Passalora fulva* (Capnodiales). Some unexpected findings included a chaetoglobosin-like BGC in *Macrophomina phaseolina.* Chaetoglobosins are a class of mycotoxins with both antifungal and anticancer activities (ALi et al. 2015; Jiang et al. 2017) found in the distantly related *Chaetomium globosum* (Sordariomycetes) and some Eurotiomycetes (Schümann and Hertweck 2007) (fig. 2 and supplementary table S1, Supplementary Material online). Another unexpected finding was a leucinostatin-like cluster in *Macrophomina phaseolina.* Leucinostatin is a peptaibol compound with putative antimicrobial and antifungal activity, previously only known from Sordariomycetes taxa (Wang et al. 2016).

### Cluster Co-Occurrence Networks Reveal Contrasting Trends in Diversification

**Fig. 3.**
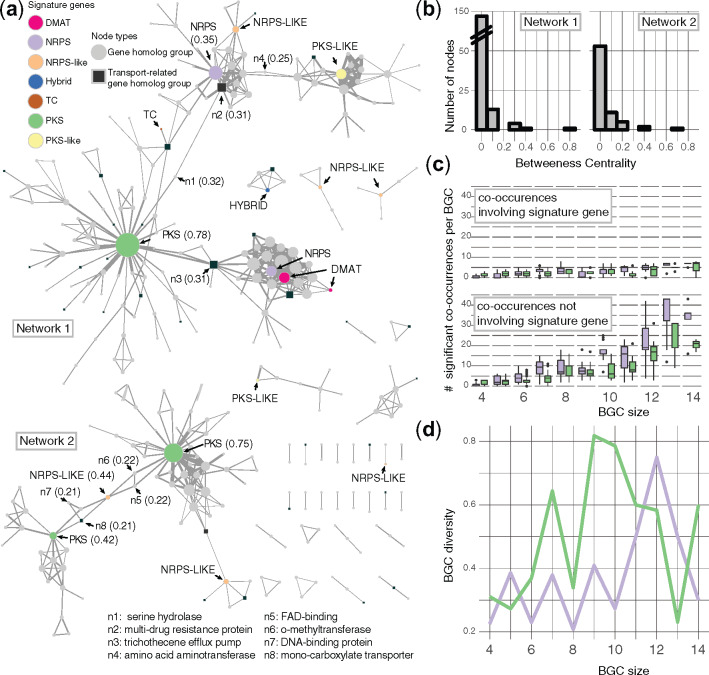
Gene co-occurrence networks among biosynthetic signature gene clusters. (*a*) Co-occurrence network of gene homolog groups. Nodes in the co-occurrence network represent all gene homolog groups found in a BGC family. Edges represent significant co-occurrences (empirical probability ≤0.05 based on the null distribution, see Materials and Methods) between gene homolog groups. Node size is proportional to the number of significant co-occurrences involving that gene homolog group, and edge width is proportional to the number of unique BGC families with ≥4 gene homolog groups that contain the co-occurrence. Distance between nodes is proportional to the number of co-occurrences they have in common, adjusted by edge width. Signature genes (colored circles) and transport-related function (squares) are indicated. Betweenness centrality scores ≥0.2 are indicated in parentheses for signature genes and eight other nodes (n1–8). Networks 1 and 2 are the two largest networks. (*b*) Histogram of betweenness centrality scores for all nodes in networks 1 and 2 (bin width = 0.1). (*c*) Significant co-occurrences within PKS and NRPS clusters. Boxplots of gene homolog group co-occurrences involving signature genes (top) and nonsignature genes (bottom) across all polyketide synthase (PKS; green) and nonribosomal polypeptide synthetase (NRPS; purple) clusters with ≥4 unique gene homolog groups. Boxplots display the 75% percentile (top hinge), median (middle hinge), the 25th percentile (lower hinge), and outliers (dots) determined by Tukey’s method. (*d*) Diversity of PKS and NRPS clusters. A line chart tracks the diversity of PKS and NRPS clusters across all cluster sizes for both PKS (green) and NRPS (purple) clusters, where diversity is defined as the total number of unique BGC families divided by the total number of clusters.

A total of 33 discrete gene homolog co-occurrence networks were recovered (Materials and Methods; fig. 3*a*), with 71% of gene homolog groups located in the largest two networks. Signature genes tended to be highly connected to other gene homolog groups in two qualitatively different types of subnetworks. In one type of subnetwork, signature genes are centrally connected to diverse accessory gene homolog groups (e.g., PKS subnetworks), whereas in the other type one or more signature genes are noncentrally linked with fewer accessory gene homolog groups (e.g., the NRPS and DMAT subnetwork in network 1). By quantifying the betweenness centrality of each node (a function of the number of shortest network paths that pass through that node) within each network, we identified signature genes and several other biosynthetic enzymes, transporters, and DNA binding proteins that bridge alternate subnetworks (fig. 3*a* and *b*).

PKS BGCs are more compositionally diverse than NRPS BGCs. BGCs containing PKS signature genes tended to have fewer significant co-occurrences among their constituent genes across various BGC sizes, compared with BGCs containing NRPS signature genes (fig. 3*c*). This is consistent with a trend in which PKS BGCs are more diverse across cluster sizes, compared with NRPS BGCs (fig. 3*d*).

### Different Algorithms Annotate Overlapping and Complementary Sets of Clustered Genes

**Fig. 4.**
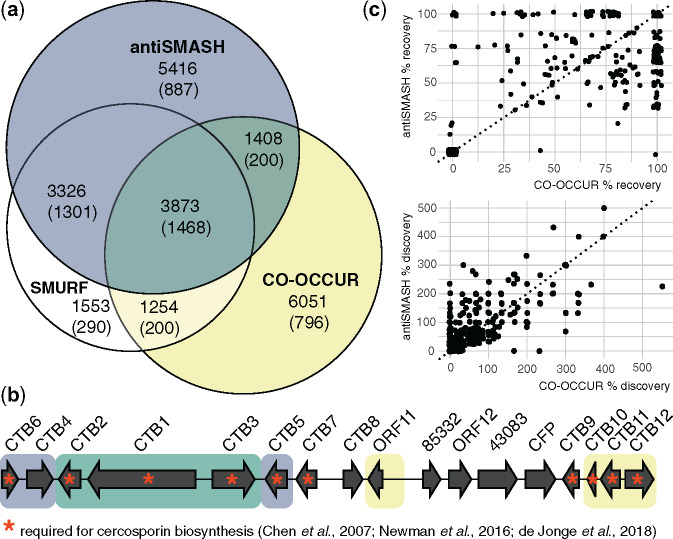
Benchmarking three different algorithms for BGC detection. (*a*) Proportional Venn diagram of distinct and overlapping BGC genes of interest detected by SMURF, antiSMASH, and CO-OCCUR. SMURF and antiSMASH use pHMMs to identify clustered genes of interest, whereas CO-OCCUR uses linkage-based criteria (see Materials and Methods). Clustered genes (unparenthesized) and secondary metabolism biosynthesis, transport, and catabolism-clustered genes (fuNOG) detected are indicated for each algorithm/combination. (*b*) Complementary recovery of the cercosporin BGC using antiSMASH and CO-OCCUR. Shading of genes in the *Cercospora zeae-maydis* cercosporin BGC (MIBiG ID BGC0001541; recovered clusterID Cerzm1_BGC0001541_h92 in supplementary table SG, Supplementary Material online) indicates genes identified by antiSMASH (blue), CO-OCCUR (yellow), or both algorithms (green). Gene names are as in de Jonge et al. (2018) and those required for cercosporin biosynthesis (Chen et al. 2007; Newman and Townsend 2016; de Jonge et al. 2018) are indicated with an asterisk. (*c*) Gene recovery and discovery in clusters homologous to known BGCs. Scatterplots show the percent of genes recovered (top) or discovered (bottom) by antiSMASH versus CO-OCCUR at each locus homologous to a MIBiG BGC (search criteria: minimum three-gene cutoff; minimum of 75% genes similar to MIBiG BGC genes in locus). Percent recovery is defined as the number of genes identified by BlastP in an algorithm-identified cluster divided by the size of the BlastP identified BGC, multiplied by 100. Percent discovery is defined as the number of genes identified by the cluster algorithm but not identified in the BlastP search, divided by the size of the BlastP identified BGC, multiplied by 100. *y* = *x* at the dotted reference line.

CO-OCCUR predictions and the profile Hidden Markov Model (pHMM)-based SMURF (Khaldi et al. 2010) and antiSMASH (Blin et al. 2017) programs all predicted similar absolute numbers of BGCs with four or more gene homolog groups, but these BGCs varied in their predicted content. antiSMASH identified a total of 1,710 BGCs that were part of 252 BGC families and 887 BGC singletons, occurring in only one genome (supplementary tables SH and SI, Supplementary Material online). SMURF identified a total of 686 BGCs that were part of 194 BGC families and 495 BGC singletons (supplementary tables SJ and SK, Supplementary Material online). CO-OCCUR predicted 1,469 BGCs with four or more gene homolog groups that are part of 239 BGC families and 220 BGC singletons (supplementary tables SC and SE, Supplementary Material online). We found that no single algorithm was able to annotate all predicted genes of interest in a BGC, even those predicted to be involved in SM biosynthesis (fig. 4*a* and supplementary table SL, Supplementary Material online). CO-OCCUR identified 51.2% and 37.7% of the clustered genes detected by SMURF and antiSMASH, respectively. Conversely, SMURF and antiSMASH identify 40.7% and 42.0% of the clustered genes detected by CO-OCCUR, respectively. When examining only genes predicted to participate in SM biosynthesis, transport, and catabolism, we found that CO-OCCUR identified 51.2% and 43.3% of genes detected by SMURF and antiSMASH, respectively, whereas SMURF and antiSMASH each identified 62.6% of those detected by CO-OCCUR (fig. 4*a*).

The complementary nature of the CO-OCCUR and antiSMASH algorithms is illustrated by their annotations of a characterized BGC that encodes the biosynthesis of cercosporin (fig. 4*b*), a nonhost-specific polyketide produced by *Cercospora* spp. (*Dothideomycetes*) and *Colletotrichum* (Sordariomycetes) (de Jonge et al. 2018). Encoded in a BGC, all ten genes involved in cercosporin biosynthesis are known and characterized (*CTB1-3*, *CTB5-7*, and *CTB9*), in addition to a regulator (*CTB8*) and two transporters (*CTB4* and *CFP*) (Chen et al. 2007; Newman and Townsend 2016; de Jonge et al. 2018). At this BGC’s locus in *Cercospora zeae-maydis*, both antiSMASH and CO-OCCUR annotated *CTB1*, *CTB2*, and *CTB3* as genes of interest; only antiSMASH annotated *CTB4*, *CTB5*, and *CTB6*; only CO-OCCUR annotated *CTB10*, *CTB11*, and *CTB12*; and no algorithm annotated *CTB7*, *CTB8*, *CTB9*, or *CFP*.

CO-OCCUR and antiSMASH recovered similar proportions of loci homologous to known BGCs and both predicted additional genes of interest in the vicinity of these candidates. We identified 364 BGCs with ≥3 genes across all *Dothideomycetes* genomes that are homologous to 58 characterized BGCs from the MIBiG database (supplementary table SM, Supplementary Material online). We found that both antiSMASH and CO-OCCUR recovered similar percentages of BGC content (antiSMASH mean percent recovery = 48.3%, SD = 37.6%; CO-OCCUR mean percent recovery = 51.0%, SD = 42.6%), although for any given BGC, percent recovery often differed between each algorithm (fig. 4*c* and supplementary table SG, Supplementary Material online). Large standard deviations in recovery scores likely reflect differential likelihood of identifying genes “of interest” among BGCs. We also found that both antiSMASH and CO-OCCUR identified similar numbers of new genes of interest around BGC loci (antiSMASH mean percent discovery = 65.4%, SD = 85.4%; CO-OCCUR mean percent discovery = 56.6%, SD = 89.4%). The number of additional genes of interest often exceeded the size of the recovered candidate cluster resulting in percent discovery values >100%, which in turn resulted in high standard deviations. High rates of novel gene discovery might reflect the incomplete annotation of many clusters in MIBiG.

### Some Overdispersed Clusters Have Ecologically Biased Distributions

**Fig. 5.**
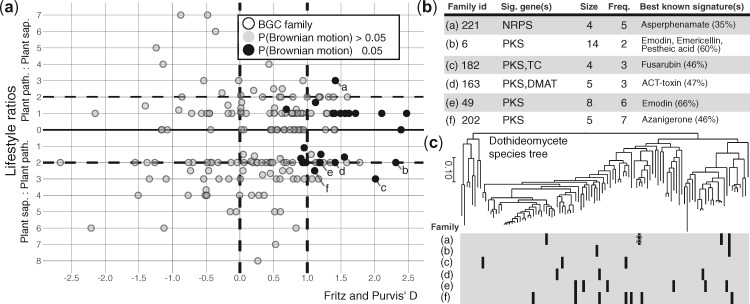
Phylogenetic and ecological signal in the distributions of BGC families. (*a*) Scatterplot of phylogenetic and ecological signal of BGC families. Values along the *x*-axis correspond to Fritz and Purvis’ *D* statistic, representing phylogenetic signal in a BGC family’s distribution and values along the *y*-axis indicate the absolute ratios of lifestyles (pathotroph/saprotroph above and saprotroph/pathotroph below the *x*-axis). Distributions of BGC families with *D* < 0 are more conserved compared with a Brownian model of trait evolution, and distributions of BGC families with *D* > 1 are considered overdispersed. Points representing BGC family distributions with probability of Brownian trait evolution (P(Brownian)) ≤0.05 are in black, whereas those >0.05 are in gray. BGC families with P(Brownian) ≤0.05 and a lifestyle ratio ≥2 are labeled and described in (*b*) and (*c*). Only BGC families with ≥4 unique gene homolog groups per cluster are shown. (*b*) Summary descriptions of labeled BGC families. Sig. genes, signature genes present in the BGC family; Size, number of unique gene homolog groups in the BGC family reference cluster; Freq., number of fungi with a cluster that belongs to the BGC family; and Best known signature(s), signature gene(s) from the MIBiG database with the highest similarity to signature genes from the BGC family, with average percentage similarity shown in parentheses. (*c*) Phylogenetic distributions of labeled BGC families. Presence (black cells) and absence (gray cells) matrix of clusters assigned to each labeled BGC family across *Dothideomycetes* genomes tree as in figure 2.

**Fig. 6.**
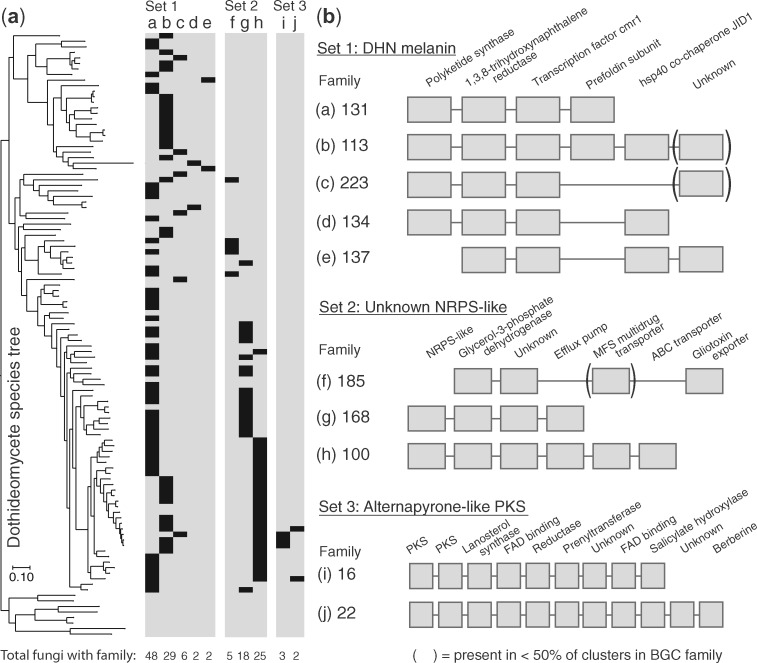
Three examples of BGC families with conserved phylogenetic distributions. (*a*) BGC family distributions. Presence (black cells) and absence (gray cells) matrix of clusters assigned to various BGC families (columns a–j, described in part *b*), across *Dothideomycetes* genomes tree as in figure 2. Each matrix contains distinct sets of BGC families that are separated by ≤0.05 distance units on the complete linkage tree in figure 2. The number of fungi with each BGC family is indicated at the bottom of each column. (*b*) BGC family composition. BGC families in set 1 are predicted to encode DHN melanin biosynthesis and is found in nearly all *Dothideomycetes*; set 2 contains unknown BGC families with NRPS-like signature genes and is restricted to Pleosporales; set 3 contains unknown BGC families with PKS signature genes, where the PKSs from group 16 are on average 84% similar to the PKS in the characterized alternapyrone cluster (MIBiG ID: BGC0000012), and is found in *Bipolaris* and *Dothidotthia*, two closely related genera within the Pleosporales. Gene homolog group presence in a given BGC family is indicated by a gray box below the description. Parentheses surround gene homolog groups present in <50% of clusters assigned to a given BGC family.

Using Fritz and Purvis’ *D* (Fritz and Purvis 2010), a measure of statistical dependence among species’ trait values due to their shared phylogenetic history (see Materials and Methods), we found that nearly one-fifth (18%) of BGC families with at least four genes are phylogenetically overdispersed (i.e., *D* > 1) (fig. 5 and supplementary fig. SD, Supplementary Material online). By comparison, 22.5% of BGC families had distributions that were phylogenetically underdispersed (*D* < 0). The remaining BGC family distributions either fell on the continuum between Brownian and random inheritance models (35.1%) or were present in too few taxa to be analyzed (23.8%). Six overdispersed BGC families were 2-fold overrepresented in either plant pathotrophs or plant saprotrophs. Figure 6 presents three examples of sets of highly similar BGC families, including the DHN melanin families, that vary in their phylogenetic conservation (See Materials and Methods).

### *Dothideomycetes* Have Five Families of DHN Melanin BGCs

We detected five BGC families with distinct but overlapping compositions that appear to encode partial pathways for DHN melanin biosynthesis in 87 of the 101 genomes (fig. 6 and supplementary fig. SE, Supplementary Material online). No genome had more than one predicted DHN melanin cluster. The two most prevalent families, BGC family 131 and BGC family 113, are found in 48 fungi from 10 of the 13 taxonomic orders and in 29 fungi from 6 orders, respectively. In addition to the gene homolog groups known to participate in DHN melanin biosynthesis, we detected three additional gene homolog groups (Prefoldin subunit, Heat-shock protein 40 cochaperone JID1, and a protein of unknown function) that are broadly conserved within DHN melanin clusters but that have no known role in melanin biosynthesis. As an example of how CO-OCCUR is not constrained by a priori assumptions of pHMMs, these additional gene homolog groups were not detected by either antiSMASH or SMURF despite their prevalent linkage to the known biosynthetic genes.

### SM Cluster Diversity Is Undersampled and Increases Proportionally with Total Number of Genomes in Pleosporales

**Fig. 7.**
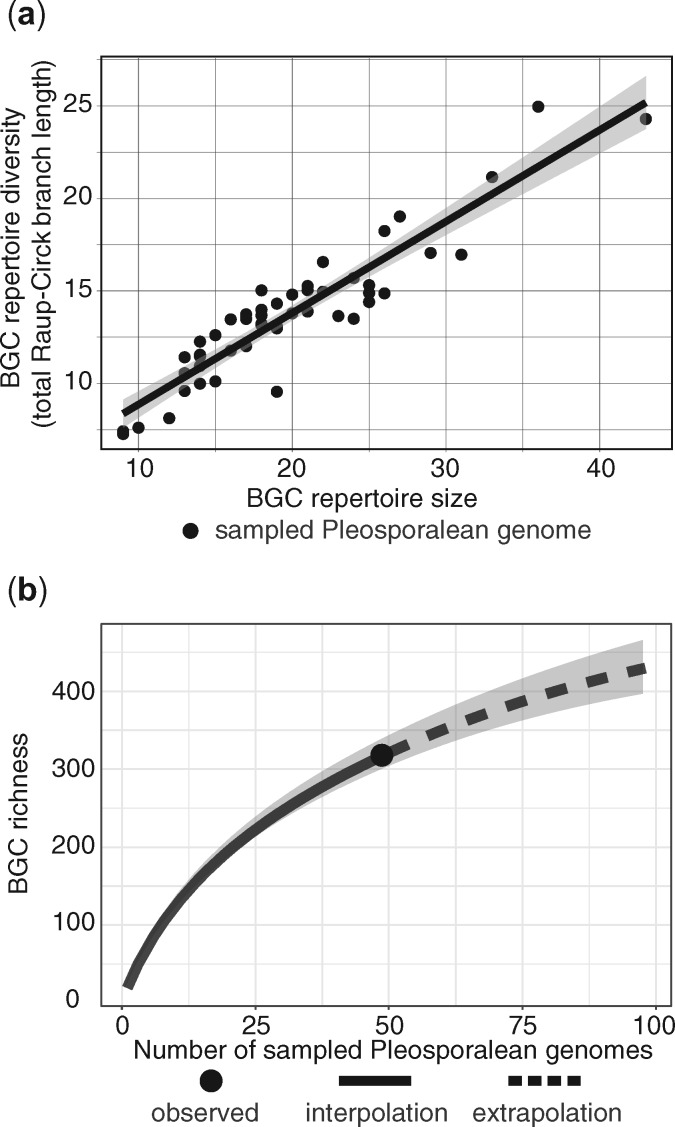
Diversity of SM gene BGC repertoires in Pleosporalean fungi. (*a*) Relationship between BGC repertoire size and BGC repertoire diversity. BGC repertoire diversity was calculated for each genome by finding the total branch length on the Raup–Crick dissimilarity tree in (*a*) associated with the set of clusters found in that genome. BGC repertoire diversity is thus a measurement of a given genome’s repertoire diversity, in terms of the gene content of its clusters. A solid line models the linear relationship between repertoire size and diversity (adj. *R*^2^ = 0.855). The shaded area around the line represents the 95% confidence interval associated with the model. (*b*) Sampled and projected SM cluster richness within the Pleosporales. Rarefied (solid lines) and extrapolated (dotted lines) estimates of SM gene cluster richness (i.e., the number of unique BGC families) with respect to the number of sampled genomes are shown for the Pleosporales. Shaded areas represent the 95% confidence intervals for both estimate types, derived from 100 bootstrap replicates. All three graphs were generated using data from the 318 unique BGC families with ≥4 unique gene homolog groups that are associated with 47 Pleosporalean fungi and 2 as yet unclassified fungi found within the Pleosporalean clade on the phylogenomic species tree in figure 2.

BGC repertoires (combinations of BGC families found within a given genome) differ markedly between fungi from different genera (mean pairwise Sørensen dissimilarity = 0.79, SD = 0.12) and to a lesser extent within a genus (mean = 0.37, SD = 0.13), with dissimilarity increasing linearly with phylogenetic distance across all pairwise species combinations among 49 Pleosporales (*y* = 0.84*x* + 0.51; *R*^2^ = 0.50), the most well-sampled *Dothideomycetes* order (supplementary figs. SF and SG, Supplementary Material online). However, given the same level of within-repertoire alpha diversity and total gamma diversity across all repertoires, dissimilarity between repertoires (i.e., beta diversity) can result from some combination of nestedness, where some repertoires are subsets of others, or turnover, where no repertoire is a subset of the other. When we partitioned total Sørensen dissimilarity between all BGC repertoires (*β*_SOR_ = 0.969) into its nestedness and turnover components, nearly all of the differences between the BGC repertoires of different genomes were due to turnover (*β*_SIM_ = 0.96) and not repertoire nestedness (*β*_SNE_ = 0.008), such that any given BGC repertoire contains a unique combination of clusters (supplementary fig. SH, Supplementary Material online). Furthermore, the compositional diversity of gene clusters within a given repertoire (supplementary fig. SF, Supplementary Material online) scales linearly with repertoire size (*y* = 0.49*x* + 3.92; adj. *R*^2^ = 0.86), indicating that clusters added to a given repertoire are generally dissimilar to the clusters already present in that repertoire (fig. 7*a*). Finally, rarefaction analysis suggests genomes within Pleosporales are undersampled with respect to BGC diversity and project substantially more unique BGC families arising from future genome sampling within this order (fig. 7*b*).

## Discussion

BGC diversity has been investigated primarily in bacteria (Cimermancic et al. 2014) and within individual genera in the fungal classes Eurotiomycetes and Sordariomycetes (Lind et al. 2017; Theobald et al. 2018; Villani et al. 2019). Although *Dothideomycetes* are producers of a number of SMs important to fungal-plant interactions and toxin production, to date there has not been a systematic evaluation of BGC diversity in the *Dothideomycetes* nor in any other fungal class. Fungal genomes experience frequent reorganization and changes in gene composition that underlies large-scale differences in chromosomal macro- and microsynteny among species (Hane et al. 2011; Grandaubert et al. 2014; Shi‐Kunne et al. 2018). Yet despite the overall dynamic nature of fungal chromatin, tight linkage is often maintained between loci with related metabolic functions, manifesting as gene clusters (Del Carratore et al. 2019). Here, we developed an alternative, function-agnostic approach to annotating SM genes of interest that exploits these patterns of microsynteny in order to identify previously unexplored dimensions of fungal BGC diversity.

### Complementary Methodologies Enhance Understanding of BGC Composition and Diversity in *Dothideomycetes*

There are two main approaches to predicting genes that are functionally associated in BGCs. The first uses targeted methods based on precomputed pHMMs derived from a set of genes known to participate in SM metabolism to identify sequences of interest (Khaldi et al. 2010; Blin et al. 2017). The second uses untargeted methods based on some function-agnostic criteria, such as synteny conservation or shared evolutionary history, to implicate genes as part of a gene cluster (Gluck-Thaler and Slot 2018). Due to common metabolic functions employed across distantly related taxa, targeted approaches, such as those employed by SMURF and antiSMASH, have proven enormously successful. However, our objective in this study was to develop a complementary untargeted approach in order to capture undescribed BGC diversity within a single fungal lineage.

Our CO-OCCUR algorithm leverages a database of 101 *Dothideomycetes* genomes in order to annotate genes of interest using unexpectedly conserved genetic linkage as an indicator of selection for coinheritance with SM signature genes. CO-OCCUR failed to recover many of the genes annotated using the pHMM approaches employed by SMURF and antiSMASH, indicating that it has limitations in its prediction of SM BGC content. These results suggest that it is not optimal for the de novo BGC annotation of individual genomes, and its ability to annotate genes of interest is proportional to their co-occurrence frequency in a given database, meaning that it is not well suited for recovering associated SM genes that are not evolutionarily conserved. This may explain in part why 10,295 genes (including 2,478 genes predicted to be involved in secondary metabolism) identified by antiSMASH and SMURF combined were not detected with CO-OCCUR (fig. 4), and why CO-OCCUR detected only a few of the host-selective toxins found in *Dothideomycetes*.

Nevertheless, our method avoids some of the limitations intrinsic to algorithms that employ pHMMs to delineate cluster content. Although pHMM-based approaches gain predictive power by leveraging similarities in SM biosynthesis across disparate organisms, they may fail to identify gene families involved in secondary metabolism that are unique to a particular lineage of organisms. For example, SMURF detects accessory SM genes using pHMMs derived from mostly *Aspergillus* (Eurotiomycetes) BGCs (Khaldi et al. 2010), whereas antiSMASH v4 and v5 use 301 pHMMs of smCOGs (secondary metabolism gene families) derived from aligning SM-related proteins, of which few are currently from fungi, in order to identify genes of interest in the regions surrounding signature biosynthetic genes (Blin et al. 2017). Taxonomic bias introduced by sampling a limited number of BGCs may account for the 6,051 proteins found in BGCs that were identified by CO-OCCUR but not any other algorithm, of which 796 are predicted to participate in secondary metabolism and 617 could not be assigned to a COG category but nevertheless have domains commonly observed in SM biosynthetic proteins (e.g., methyltransferase, hydrolase).

A linkage-based approach can also identify noncanonical accessory genes involved in SM biosynthesis. For example, we detected three genes among the five variants of the DHN melanin cluster that were not previously considered to be part of this BGC and not detected by either antiSMASH or SMURF. One of these genes, a predicted HSP40 chaperone, is a homolog of the yeast gene JID1, whose knock-out mutants display a range of phenotypes (https://www.yeastgenome.org/locus/S000006265/phenotype, last accessed May 27, 2020) related to melanin production, including increased sensitivity to heat and chemical stress. We propose that natural selection (not genetic hitchhiking) is responsible for conservation of synteny in these loci, because SM cluster locus composition and microsynteny in general are typically highly dynamic in fungi (Lind et al. 2017; Proctor et al. 2018), and therefore conserved linkage in these clusters over speciation events is a strong indicator of related function (de Jonge et al. 2018; Del Carratore et al. 2019). The identification of genes with noncanonical functions, including those not participating directly in SM biosynthesis, may reveal SM supportive functions, including mechanisms to protect endogenous targets of the metabolic product (Keller 2015), in addition to novel biosynthetic genes (de Jonge et al. 2018).

Ultimately, targeted and untargeted approaches to BGC annotation reinforce and enrich our understanding of BGC diversity, as no single method identifies all accessory genes of interest in the regions surrounding signature biosynthetic genes (fig. 4). It is notable that the cercosporin BGC was long thought to consist only of *CTB1*-8, based on functional analyses and structural prediction. However, de Jonge et al. recently predicted *CTB9*-12 to be of interest after observing that these genes have conserved synteny among all fungi that possessed *CTB1*-8 and subsequently demonstrated they are essential for cercosporin biosynthesis (de Jonge et al. 2018). Only CO-OCCUR detected these additional four genes and both pHMM-based models and CO-OCCUR were required to detect the complete cercosporin BGC in our study. Given the complementary nature of the advantages and disadvantages of different algorithms, we suggest future studies incorporate multiple lines of evidence from both targeted and untargeted approaches to more fully capture BGC compositional diversity. The 332 gene homolog groups of interest that we identified using CO-OCCUR could further be used to build pHMMs and be incorporated into existing BGC annotation pipelines in order to facilitate more complete analyses of single genomes.

### Signature Genes Differ in Mode of BGC Diversification

Although BGCs in fungi typically display characteristics of diversification “hotspots,” showing elevated rates of gene duplication and gene gain and loss (Wisecaver et al. 2014; Lind et al. 2017), modular parts of clusters and even entire clusters are often shared between divergent species. BGC diversification through gain and loss of individual genes and subclusters of genes has been demonstrated in bacterial BGC diversity (Cimermancic et al. 2014). Although the extent of subclustering in fungal genomes has never been directly addressed to our knowledge, the algorithm we designed here essentially functions by identifying the smallest possible type of subcluster: A pair of genes found more often than expected by chance. The unexpected co-occurrence of gene pairs revealed that the two largest types of signature gene families, PKS and NRPS, have contrasting co-occurrence network structures. NRPS gene homolog groups are embedded in highly reticulate cliques (i.e., form unexpected associations with genes that co-occur amongst themselves). This could suggest NRPS cluster diversification is constrained by interdependencies among accessory genes. By contrast, PKS gene homolog groups are network hubs (i.e., form unexpected associations with many non-co-occurring genes), which may underlie the higher compositional diversity and decreased frequency of unexpected co-occurrences found within PKS clusters (fig. 3*c* and *d*). The apparent contrast in how these different signature BGC families are assembled may reflect the range of accessory modifications typically applied to the structures of polyketides and nonribosomal peptides produced by PKSs and NRPSs. Alternatively, PKS clusters may be subject to more diversifying selection, due to the ability of cognate metabolism in other organisms to utilize, degrade, or neutralize the metabolites. These hypotheses remain to be tested.

### Persistent Gene Co-Occurrences Reveal Layers of Combinatorial Evolution

Previous large-scale analyses of BGCs suggest that there is an upper limit to the number of gene families that associate with signature biosynthetic genes, and that diversity is in large part dependent on combinatorial reshuffling of existing loci (Cimermancic et al. 2014). Our analysis expands the number of gene families implicated in BGC diversity and identifies patterns of modular combinatorial evolution among accessory gene homolog groups with metabolic, transport, and regulatory-related functions. Although some of these accessory gene homolog groups are restricted to BGCs with a particular type of signature SM gene, others are present in multiple BGC with different signature SM genes, suggesting they encode evolvable or promiscuous functions that can be readily incorporated into different metabolic processes (supplementary table SB, Supplementary Material online). For example, 34 gene homolog groups with predicted transporter functions are common features of the clusters we detected, present in just under half (43%) of all predicted clusters. Among these gene homolog groups, five (MCL000003, MCL000005, MCL000016, MCL000193, and MCL000109) have been recruited into especially diverse BGCs and are primarily annotated as toxin efflux transporters or multidrug resistance proteins. These five transport-related genes are found in 33% of all BGC families, and in at least one BGC in 99% of all examined species. Transporters are a key component of fungal chemical defense systems, well known for facilitating resistance to fungicides and host-produced toxins (Coleman and Mylonakis 2009). Transporters are also increasingly recognized as integral components of self-defense mechanisms against toxicity of endogenously produced SMs (Menke et al. 2012).

### Heterogeneous Dispersal Patterns of BGCs Underpin Fungal Ecological Diversity

The distribution of fungal chemodiversity remains difficult to observe and interpret directly, making BGCs useful tools for elucidating underlying trends in fungal chemical ecology. Although the vast majority of BGCs remain uncharacterized, their phylogenetic distributions occasionally provide clues to the selective environments that promote their retention (Slot 2017). For example, spotty distributions resulting from horizontal transfer of BGCs between distantly related but ecologically similar species suggest that the encoded metabolites contribute to fitness in the shared environment (Dhillon et al. 2015; Reynolds et al. 2018). Shared ecological lifestyle may also help explain why certain clusters, such as those involved in putative degradative pathways, are retained among phylogenetically distant species (Gluck-Thaler and Slot 2018). Our simple ecoevolutionary screen identified 43 BGCs that are more widely dispersed than expected under neutral evolutionary models and further revealed that a subset of these BGCs are present more often in fungi with specific nutritional strategies (e.g., plant saprotrophs and plant pathotrophs), suggesting the molecules they encode contribute to specific plant-associated lifestyles (fig. 5). For example, we found an overdispersed NRPS BGC (family 221) that is present in three plant pathogens and one plant saprotroph. In contrast, the 54 BGCs showing a phylogenetically underdispersed distribution among mostly closely related genomes is consistent with lineage-favored traits, which may or may not be due to shared ecology. For example, a monophyletic clade of 26 pleosporalean fungi all have a six-gene NRPS-like cluster (family 100) of unknown function, fully maintained among these allied taxa (and a single distant relative), suggesting it encodes a trait that contributes to the success of this lineage. Phylogenetic screens, especially when coupled with more robust phylogenetic analyses, such as gene tree-species tree reconciliation methods and hypothesis testing using phylogenies representing alternative evolutionary scenarios, will be useful for prioritizing the characterization of BGCs most likely to contribute to the success of particular guilds or clades.

Among those BGCs with hits to the MIBiG database, we identified clusters that displayed both lineage-specific and spotty or sporadic distributions. The Pleosporales, for example, contains many plant pathogens and the conservation of BGCs involved in production of general virulence factors toward plants such as solanopyrone, alternapyrone, and the extracellular siderophore dimethylcoprogen across many taxa in this order suggests a shared lineage-specific trait with roles in plant pathogenesis. In contrast, the aflatoxin-like cluster Dothistromin cluster, which was proposed to be horizontally transferred from *Aspergillus* (Eurotiomycetes), had a very spotty distribution, found only in several closely related taxa in Capnodiales, supporting a hypothesis of HGT (Bradshaw et al. 2013). Similarly, the ETP toxin sirodesmin shares six genes with the BGC producing the ETP toxin gliotoxin, which plays a role in virulence toward animals in human pathogen *Aspergillus fumigatus* (Eurotiomycetes) (Gardiner et al. 2004; Bok et al. 2006). Related ETP-like BGCs have since been identified in a number of other taxa of Eurotiomycetes and Sordariomycetes, but among *Dothideomycetes* were previously known only from *L. maculans* and a partial cluster in *Sirodesmin diversum* lacking the core NRPS (Patron et al. 2007)*.* We detected homologs of this cluster found sporadically distributed in several other taxa within Pleosporales (fig. 2 and supplementary table SG, Supplementary Material online). The CO-OCCUR algorithm detected only a few BGC with hits to host-selective toxins (sirodesmin and T-toxin) but failed to detect several well-known host-selective toxins such as HC-toxin and other host-selective toxins in *Alternaria alternata*. Either these host-selective toxins are not represented in MIBiG or as discussed above, the uniqueness of these clusters and rarity of the linkages between genes in these clusters in the overall data set may make them difficult to detect through CO-OCCUR.

### Variation among BGC Repertoires Is Due to High BGC Turnover, Not Nestedness

Recent comparative studies have documented high intraspecific diversity of SM pathways within and between different species of plants, bacteria, and fungi (Penn et al. 2009; Holeski et al. 2012, 2013; Choudoir et al. 2018; Vesth et al. 2018). However, identical estimates of diversity can result from two distinct processes: nestedness, where one set of features is entirely subsumed within another, or turnover, where differences are instead due to a lack of overlap among the features of different sets (Baselga 2012). When we partitioned diversity among BGC repertoires in Pleosporales (i.e., beta diversity), we found that the vast majority of variation is due to a high degree of genome-specific cluster combinations, and not nestedness (fig. 7 and supplementary fig. SH, Supplementary Material online). Much of the turnover in BGC repertoire content between genomes appears to occur over relatively short evolutionary timescales (supplementary fig. SF, Supplementary Material online) and then diversifies more gradually, suggesting that divergence in repertoires may be closely linked to speciation processes, such as niche differentiation or geographic isolation. Directional selection, especially for multigenic traits encoded at a single locus (e.g., BGCs), leads to rapid gain/loss dynamics exemplary of many SM phenotypes and genotypes (Lind et al. 2017; Choudoir et al. 2018). Niche differentiation further reinforces divergence between closely related repertoires, which might lead to rapid accumulation of variation over short evolutionary timescales. Indeed, evidence from within populations suggests that BGCs are occasionally located in genomic regions experiencing selective sweeps in geographically isolated pathogen populations (Hartmann et al. 2018). The retention/loss of certain SM clusters is coincident with speciation in bacteria (Kurmayer et al. 2015) and much of the variation in BGC repertoires in *Metarhizium* insect pathogens is species specific (Xu et al. 2016). Within *Dothideomycetes*, the evolution of host-selective toxins even within a single species of pathogen, for example, may allow for niche differentiation, host specialization, and potentially speciation. Rare chemical phenotypes, especially with regards to defense chemistry, may also increase fitness in complex communities (Kursar et al. 2009).

### BGCs Interact with Dimensions of Chemical Diversity

Biological activity of a SM can increase organismal fitness, but any given molecule is not likely to be biologically active. The screening hypothesis posits that mechanisms to generate and retain biochemical diversity would therefore be selected, despite the energetic costs, because increasing structural diversity increases the probability of “finding” those that are adaptive (Firn and Jones 2003). This phenomenon is analogous to the mammalian immune system’s latent capacity to generate novel antibodies, resulting in a remarkable ability to respond to diverse antagonists (Firn and Jones 2003). However, although the screening hypothesis may equally apply to plants and microorganisms, patterns of diversity we observe here suggest that each lineage generates and maintains biochemical diversity in fundamentally distinct ways. Specifically, fungal individuals appear to maximize total chemical beta diversity while simultaneously minimizing alpha diversity of similar chemical classes (Nielsen et al. 2017; Vesth et al. 2018). In contrast, individual plants are more likely to produce diverse suites of structurally similar molecules (Li et al. 2015; Luu et al. 2017; Song et al. 2017). We show that total cluster diversity increases linearly with repertoire size across a broad sample of fungi, extending previous observations that individual fungal genomes are streamlined to produce molecules that share little structural similarity. Rather than maintaining sets of homologous BGCs and pathways within the same genome, evidence from ours and other studies suggests that fungi instead maintain high genetic variation in homologous BGCs across individuals at the level of the pan-genome (Ziemert et al. 2014; Lind et al. 2017; Olarte et al. 2019). Although not a selectable evolvability mechanism per se, greater access to the diversity of BGCs harbored in pan-genomes through recombination, hybridization, and horizontal transfer effectively outsources the incremental screening for bioactive metabolites across many individuals, thereby decreasing the costs for generating diversity for any given individual and likely accelerating the rate at which effective bioactive metabolite repertoires are assembled within a given lineage (Slot and Gluck-Thaler 2019). Our characterization of BGC diversity across the largest fungal taxonomic class represents a step toward elucidating the broader consequences of these contrasting strategies for generating and maintaining biodiversity of metabolism writ large.

## Conclusions

Fungi produce a range of SMs that are linked to different ecological functions or defense mechanisms, playing a role in adaptation over time. Although studied at intra- and interspecific level, this phenomenon has not been studied at macroevolutionary scales. The *Dothideomycetes* represent the largest and phylogenetically most diverse class of fungi, displaying a range of fungal lifestyles and ecologies. Here, we assessed the patterns of diversity of BGCs across the genomes of 101 *Dothideomycetes* to dissect patterns in the evolution of chemodiversity. Our results suggest that different classes of BGCs (e.g., PKS vs. NRPS) have differing diversity of cluster content and connectedness among networks of co-occurring genes and implicate high rates of BGC turnover, rather than nestedness, as the main contributor to the high diversity of BGCs observed among fungi. Consequently, little overlap was found in BGCs from different genera, consistent with diverse ecologies and lifestyles among the *Dothideomycetes*, and suggesting that most of the metabolic capacity of this fungal class remains to be discovered.

## Materials and Methods

### *Dothideomycetes* Genome Database and Species Phylogeny

A database of 101 *Dothideomycetes* annotated genomes, gene homolog groups, and the corresponding phylogenomic species tree were obtained from Grigoriev et al. (2014) and Haridas et al. (2020).

### Gene Cluster Annotation with the SMURF Algorithm

We used a command-line Python script based on the SMURF algorithm (Vesth et al. 2018). Using genomic coordinate data and annotated PFAM domains of predicted genes as input, the algorithm predicts seven classes of SM clusters based on the multi-PFAM domain composition of known “backbone” genes. The BGC classes are 1) polyketide synthases (PKSs), 2) PKS-like, 3) NRPSs, 4) NRPS-like, 5) hybrid PKS-NRPS, 6) prenyltransferases (DMATs), and 7) TCs. The borders of clusters are determined using PFAM domains that are enriched in characterized SM clusters, allowing up to 3 kb of intergenic space between genes, and no more than six intervening genes that lack SM-associated domains. SM-associated PFAM domains were borrowed from Khaldi et al. (2010).

### Gene Cluster Annotation with antiSMASH

All genomes were annotated using antiSMASH v4.2.0 by submitting genome assemblies and GFF files to the public web server with options “use ClusterFinder algorithm for BGC border prediction” and “smCOG analysis” (Blin et al. 2017). antiSMASH reports all genes within the borders of a predicted cluster as part of the cluster. For our analysis, we only considered genes belonging to annotated smCOGs or signature biosynthetic gene families as part of a given cluster and excluded all others, in order to obtain conservative, high confidence estimations of cluster content based on genes of interest.

### Sampling Null Gene Homolog Group Pair Distributions

We created null distributions from which we could empirically estimate co-occurrence probabilities by randomly sampling gene homolog group pairs without replacement from all *Dothideomycetes* genomes (fig. 1 and supplementary fig. SA, Supplementary Material online). Before beginning, we defined null distributions based on two parameters: a range of sizes for the smallest gene homolog group in the pair and a range of sizes for the largest gene homolog group in the pair, where each range progressively incremented by 25 from 1 to 800 and all combinations of ranges were considered. For example, there existed a null distribution for gene homolog group pairs where the smallest gene homolog group had between 26 and 50 members, and the largest gene homolog group had between 151 and 175 members. To begin, we randomly sampled a genome and then randomly selected two genes within six genes of one other from that genome. We retrieved the gene homolog groups to which those genes belonged and then counted the number of times members of each gene homolog group were found within six genes of each other across all *Dothideomycetes* genomes. We counted the number of members belonging to each gene homolog group, excluding those that were found within six genes of the end of a contig, in order to obtain a corrected size for each gene homolog group that accounted for variation in assembly quality. The co-occurrence observation was then stored in the appropriate null distribution based on the corrected sizes of each gene homolog group. For example, the number of co-occurrences of a sampled gene homolog group pair where the smallest gene homolog group had a corrected size of 89, and the largest gene homolog group had a corrected size of 732 would be placed in the null distribution where the smallest size bin was 76–100 members and the largest size bin was 726–750. All gene homolog groups with >800 members were assigned to the 776–800 size bin. This sampling procedure was repeated 500,000 times. After evaluating various bin sizes, we ultimately decided to use a range of 25 because this resulted in the most even distribution of samples across all null distributions. Due to variation in the number of gene homolog groups with any given size across our data set, it was not possible for all null distributions to contain the same number of samples.

### The CO-OCCUR Pipeline

Current BGC detection algorithms first identify signature biosynthetic genes using pHMMs of genes known to participate in SM biosynthesis and then search predefined regions surrounding signature genes for colocated “accessory” biosynthetic, regulatory, and transport genes. The approach of CO-OCCUR, in contrast, is to define genes of interest based on whether they are ever found to have unexpectedly conserved syntenic relationships with other genes in the vicinity of signature biosynthetic genes, agnostic of gene function. Here, we used CO-OCCUR in conjunction with a preliminary SMURF analysis to arrive at our final BGC annotations (fig. 1 and supplementary fig. SA, Supplementary Material online). We first took all SMURF BGC predictions and extended their boundaries to genes within a six-gene distance that belonged to gene homolog groups found in another SMURF BGC, effectively “bootstrapping” the BGC annotations in order to ensure consistent identification of BGC content across the various genomes. SMURF BGCs at this point in the analysis were considered to consist of all genes found within the cluster’s boundaries. For each pair of genes in each BGC (including signature biosynthetic genes), we retrieved their gene homolog groups and kept track of how many times that gene homolog group pair was observed across all BGCs. Then, for each observed gene homolog group pair, we divided the number of randomly sampled gene homolog group pairs in the appropriate null distribution (based on the corrected sizes of the smallest and largest gene homolog groups within the observed pair, see above) that had a number of co-occurrences greater than or equal to the observed number of co-occurrences by the total number of samples in the null distribution. In doing so, we empirically estimated the probability of observing a gene homolog group pair with at least that many co-occurrences by chance, given the sizes of the gene homolog groups. In this way, we were able to take into account the relative frequencies of each gene homolog group within a pair across all genomes when assessing the probability of observing that pair’s co-occurrence. For example, if we observed that gene homolog group 1 and gene homolog group 2 co-occurred 19 times within SMURF-predicted BGCs, and that gene homolog group 1 had 57 members, whereas gene homolog group 2 had 391 members, we would count the number of randomly sampled gene homolog group pairs that co-occurred 19 or more times within the null distribution where the smallest gene homolog group size bin was 51–75 and the largest gene homolog group size bin was 376–400, and then divided this by the total number of samples in that same null distribution to obtain the probability of observing gene homolog group 1 and gene homolog group 2’s co-occurrences by chance. All co-occurrences with an empirical probability estimate of ≤0.05 were considered significant and retained for further analysis. In order to decrease the risk of false positive error, we did not evaluate the probability of observing any gene homolog group pairs with less than five co-occurrences and also did not evaluate any gene homolog group pairs whose corresponding null distribution had fewer than 10 samples.

Next, in order to obtain our final set of predicted BGCs, we took all gene homolog groups found in significant co-occurrences and conducted a de novo search in each genome for all clusters containing genes belonging to those gene homolog groups within a six-gene distance of each other. In this way, all BGC clusters in our final set consisted of genes that belonged to these gene homolog groups of interest, whereas all other intervening genes were not considered to be part of the cluster. We treated gene homolog groups containing signature biosynthetic genes as we would any other gene homolog group: If a signature gene predicted by SMURF was not a member of a gene homolog group part of an unexpected co-occurrence, we did not consider it part of any clusters. We stress that co-occurrences were only used to determine gene homolog groups of interest, but that once those gene homolog groups were identified, they did not need to be part of an unexpected co-occurrence within a predicted cluster in order to be considered part of that cluster. By focusing only on genes that form unexpected co-occurrences, it is likely that we have underestimated the compositional diversity of *Dothideomycetes* BGCs (but this may be the case for all cluster detection algorithms; see Results).

We then grouped all predicted BGCs into BGC families based on a minimum of 90% similarity in their gene content, rounded down, in order to obtain a strict definition of BGC homology that increases the likelihood that homologous clusters encode similar metabolic phenotypes. This meant that clusters with sizes ranging from 2 to 10 were allowed to differ in at most one gene, clusters with sizes ranging from 11 to 20 were allowed to differ in at most two genes, etc. Clusters that were not at least 90% similar to any other cluster in the data set were designated as BGC singletons. Note that because there is no perfect way to determine homology when using similarity based metrics, (e.g., a ten-gene cluster could be 90% similar to a nine-gene cluster, which in turn could be 90% similar to an eight-gene cluster, but that eight-gene cluster cannot be 90% similar to the ten-gene cluster), we developed a heuristic approach for sorting clusters into groups. First, we conducted an all-vs-all comparison of content similarity to sort all clusters into preliminary groups by iterating through the clusters from largest to smallest, where size equaled the number of unique gene homolog groups, and clusters could only be assigned to a single group. Then, within each preliminary group, we identified clusters most similar to all other clusters within the group and used them as references to which all other clusters were compared during a new round of group assignment. In this final round, clusters were grouped together with a given reference into a BGC family if they were at least 90% similar to it and were classified as a BGC singleton if they were not 90% similar to any references. The often-unique composition of clusters means that in most cases, there is no ambiguity to how the clusters are classified; however, for a small number of clusters, especially those with fewer genes, there may be some ambiguity as to which group they belong.

### Annotation of BGCs and Gene Functions

In order to detect loci homologous to known BGCs in *Dothideomycetes* genomes, amino acid sequences of each annotated BGC within the MIBiG database (v1.4) were downloaded and used as queries in a BlastP search of all *Dothideomycetes* proteomes (last accessed January 4, 2019). All hits with ≥50 bit score and ≤1 × 10^−4^*e*-value were retained, and clusters composed of these hits were retrieved using a maximum of six intervening genes. In order to retain only credible homologs of the annotated MIBiG queries and to account for error in BLAST searches due to overlapping hits, we retained clusters with at least three genes that recovered at least 75% of the genes in the initial query. This set of high confidence MIBiG BGCs was then compared with the set of BGCs predicted by CO-OCCUR and antiSMASH to assess the ability of each algorithm to recover homologs to known clusters. For each algorithm and each BGC recovered using BlastP to search the MIBiG database, we calculated percent recovery, defined as the number of genes identified by the BlastP search that were also identified as clustered by the algorithm, divided by the size of the BGC identified by the BlastP search, multiplied by 100. We also calculated percent discovery, defined as the number of clustered genes identified by the algorithm but not identified in the BlastP search, divided by the size of the BGC identified by the BlastP search, multiplied by 100.

In order to annotate BGCs recovered by CO-OCCUR with characterized clusters, we used amino acid sequences of all signature biosynthetic genes in CO-OCCUR clusters as BlastP queries in a search of the MIBiG database (min. percent similarity = 70%, max *e*-value = 1 × 10^−4^, and min. high scoring pairs coverage = 50%). Basing our annotations on percent amino acid similarity to characterized signature biosynthetic genes rather than on the number of genes with similarity to BGC genes enabled a more conservative and comprehensive approach, as many BGC entries within the MiBIG database are not complete.

Proteins within predicted BGCs were annotated using eggNOG-mapper (Huerta-Cepas et al. 2017) based on fungal-specific fuNOG orthology data (Huerta-Cepas et al. 2016). Consensus annotations for all gene homolog groups were derived by selecting the most frequent annotation among all members of the group.

### Comparing BGC Detection Algorithms

In order to assess the relative performances of SMURF, antiSMASH, and CO-OCCUR, we compared all BGCs predicted by each method and kept track of the genes within those BGCs that were identified by either one or multiple methods. We summarized these findings in a Venn diagram using the “eulerr” package in R (Larsson 2018). Note that for the purposes of this analysis, BGCs predicted by SMURF and antiSMASH were considered to be composed only of genes that matched a precomputed pHMM, and BGCs predicted by CO-OCCUR were composed only of genes belonging to gene homolog groups that were part of unexpected co-occurrences, whereas all other intervening genes within the BGC’s boundaries were not considered to be part of the cluster. In doing so, we effectively ignored intervening genes that were situated between or are immediately adjacent to these clustered genes of interest for the purposes of defining a cluster’s content. Although this approach likely does not capture the full diversity of cluster composition, it is expected to decrease false positive error in BGC content prediction and represents a conservative approach to identifying what genes make up a given cluster.

### Construction of a Co-Occurrence Network

We visualized relationships between gene homolog group pairs with unexpectedly large numbers of co-occurrences in a network using Cytoscape v.3.4.0 (Shannon et al. 2003). The network layout was determined using the AllegroLayout plugin with the Allegro Spring-Electric algorithm. In order to identify hub nodes within the network, we calculated betweeness centrality, a measurement of the shortest paths within a network that pass through a given node, for each node using Cytoscape.

### Assessment of BGC Family Phylogenetic Signal

In order to quantify the dispersion of phylogenetic distributions of BGC families predicted by CO-OCCUR, we created a binary genome × BGC family matrix for all 239 BGC families with ≥4 genes that indicated the presence or absence of these BGC families across all 101 genomes. We used this matrix in conjunction with the “phylo.d” function from the “caper” package v1.0.1 in R (Orme et al. 2012) to calculate Fritz and Purvis’ *D* statistic for each BGC family’s distribution, where *D* is a measurement of phylogenetic signal for a binary trait obtained by calibrating the observed number of changes in a binary trait’s evolution across a phylogeny by the mean sum of changes expected under two null models of binary trait evolution. Fritz and Purvis’ *D* measures the degree of statistical dependence among species’ trait values due to their shared phylogenetic history. *D* is scaled such that *D* = 0 when a trait’s distribution follows a model of Brownian inheritance (where differences in trait values between species are proportional to their shared phylogenetic history), and *D* = 1 when a trait’s distribution follows a model of random inheritance (where differences in trait values between species are random with respect to their shared phylogenetic history). As *D* increases from 0 to 1, traits display increasingly random distributions; as *D* increases above 1, traits display more overdispersed distributions than the random model; conversely, as *D* decreases below 0, traits display more underdispersed or conserved distributions than the Brownian model. The first null model simulates the phylogenetic distribution expected under a model of random trait inheritance, and the second simulates the phylogenetic distribution expected under a threshold model of Brownian evolution that evolves a trait along the phylogeny where variation in that trait’s distribution accumulates at a rate proportional to branch length, such that more closely related species will have more similar trait values than distantly related species (Fritz and Purvis 2010). *D* = 0 if the trait has a phylogenetic distribution that follows the Brownian model. As values of *D* increase, the trait’s phylogenetic distribution becomes increasingly random and less dependent on branch length until reaching 1, where it has a perfectly phylogenetically random distribution. *D* > 1 if the trait has a phylogenetic distribution that is more overdispersed than the distribution of a randomly inherited trait; *D* < 0 if the trait has a phylogenetic distribution that is more conserved, or underdispersed, than the distribution of trait whose inheritance follows a Brownian model.

### Dissimilarity and Diversity Analyses

We created BGC family × gene homolog group matrices in order to determine the dissimilarity between predicted BGC families. In these matrices, for each BGC family, we indicated the presence or absence of gene homolog groups in at least one cluster within the BGC family, effectively summarizing each BGC family by integrating over the content of all clusters assigned to that family. We next used the matrix in conjunction with the “vegdist” function from the “vegan” package in R (Oksanen et al. 2016) to create a Raup–Crick dissimilarity matrix that was visualized as a dendrogram using complete linkage clustering as implemented in the “hclust” function from the core “stats” package in R. Raup–Crick dissimilarity is a commonly used statistic in community ecology to measure the probability that two sites have nonidentical species compositions. Here, we chose to use Raup–Crick dissimilarity to characterize the degree to which two BGCs have nonidentical gene homolog group compositions over other dissimilarity metrics because this statistic takes into account the global frequencies of gene homolog groups (i.e., species) sampled across all BGCs (i.e., sites), such that BGCs that share more rarely clustered gene homolog groups will appear less dissimilar than BGCs that share more commonly clustered gene homolog groups, effectively minimizing the contributions of commonly clustered gene homolog groups to overall dissimilarity. The Raup–Crick dendrograms were then used to assess the functional diversity of BGC repertoires (e.g., in the Pleosporales) by measuring the total branch distance connecting all BGC families within a given repertoire using the “treedive” function from the “vegan” package in R.

We used the same above procedure to calculate Sørensen dissimilarity between Pleosporalean genomes based on their BGC repertoires, only this time using a genome × BGC family matrix that depicted the presence or absence of BGC families across all 49 Pleosporalean genomes. Sørensen dissimilarity is a commonly used statistic in community ecology to describe the extent of overlap in species presence/absence data between two geographic sites. We applied Sørensen dissimilarity to quantify the degree of overlap in BGC family presence/absence data between two genomes. We also used this matrix to calculate and partition beta diversity in Pleosporalean BGC repertoires using the “beta.sample” function (index.family = “sorensen,” sites = 10, and samples = 999) from the “betapart” v1.4 package in R (Baselga and Orme 2012) in order to determine how much of the observed diversity among repertoires was due to gain/loss of BGC families, and how much was due to nestedness. We also used the genome × BGC family matrix to conduct a rarefaction of cluster richness across Pleosporalean genomes using the “iNEXT” function (*q* = 0, datatype = “incidence_raw,” endpoint = 98) from the “iNEXT” package in R (Hsieh et al. 2016).

## Data Availability 

All genome data are available at https://mycocosm.jgi.doe.gov/mycocosm/home and described in Haridas et al. (2020). All scripts used in the analyses are available at https://github.com/egluckthaler/co-occur. Additional data generated in this study are included in the supplementary datafile, Supplementary Material online.

Ethics Approval and Consent to Participate: No human subjects were involved in the research. 

Consent for Publication: No human data were used in the research.

## Supplementary Material

msaa122_supplementary_data
