# Supplementary material for: The Architecture of Metabolism Maximizes Biosynthetic Diversity in the Largest Class of Fungi
Source: Mol Biol Evol. 2020 May 18;37(10):2838–56. doi: 10.1093/molbev/msaa122 (PMC7530617; doi:10.1093/molbev/msaa122)

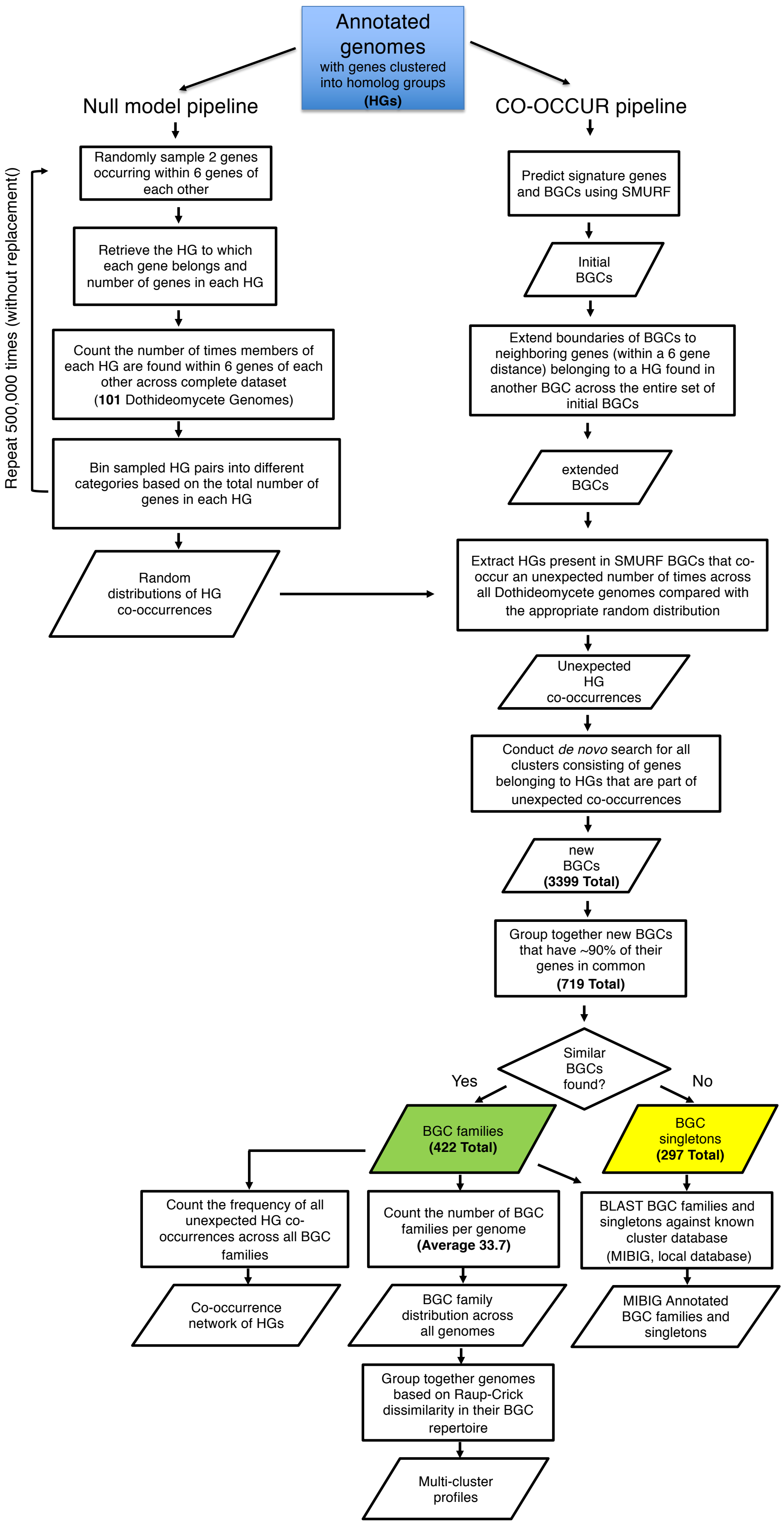
Annotated genomes
with genes clustered into homolog groups (HGs)
Null model pipeline
CO-OCCUR pipeline
Randomly sample 2 genes occurring within 6 genes of each other
Retrieve the HG to which each gene belongs and number of genes in each HG
Count the number of times members of each HG are found within 6 genes of each other across complete dataset (101 Dothideomycete Genomes)
Bin sampled HG pairs into different categories based on the total number of genes in each HG
Repeat 500,000 times (without replacement()
Random distributions of HG co-occurrences
Predict signature genes and BGCs using SMURF
Initial BGCs
Extend boundaries of BGCs to neighboring genes (within a 6 gene distance) belonging to a HG found in another BGC across the entire set of initial BGCs
extended BGCs
Extract HGs present in SMURF BGCs that co-occur an unexpected number of times across all Dothideomycete genomes compared with the appropriate random distribution
Unexpected HG co-occurrences
Conduct de novo search for all clusters consisting of genes belonging to HGs that are part of unexpected co-occurrences
new BGCs (3399 Total)
Group together new BGCs that have ~90% of their genes in common (719 Total)
Similar BGCs found?
Yes
No
BGC families (422 Total)
BGC singletons (297 Total)
Count the frequency of all unexpected HG co-occurrences across all BGC families
Co-occurrence network of HGs
Count the number of BGC families per genome (Average 33.7)
BGC family distribution across all genomes
Group together genomes based on Raup-Crick dissimilarity in their BGC repertoire
Multi-cluster profiles
BLAST BGC families and singletons against known cluster database (MIBIG, local database)
MIBIG Annotated BGC families and singletons

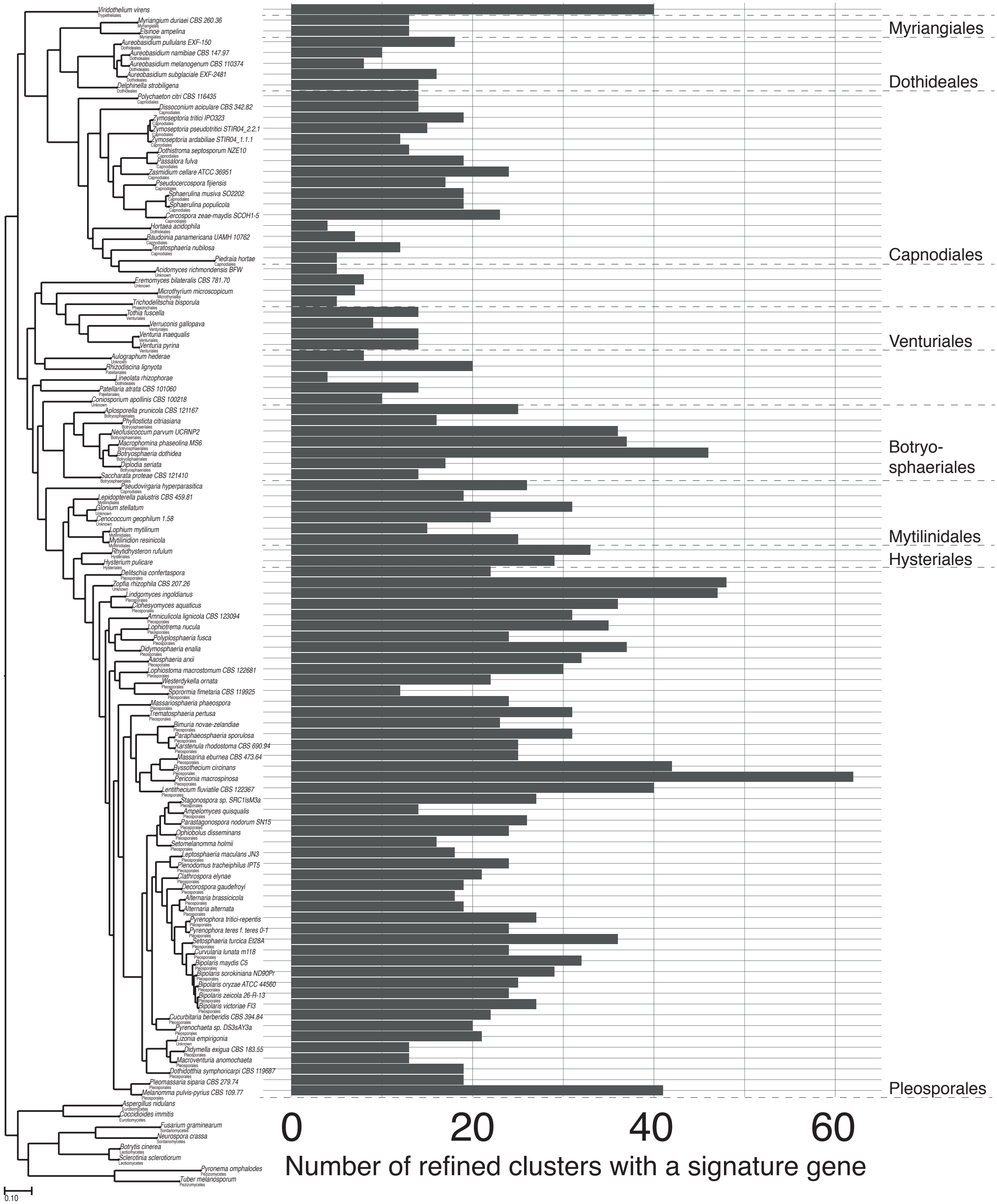

Viridothelium virens
Myriangium duriaei CBS 260.36
Elsinoe ampelina
Aureobasidium pullulans EXF-150
Aureobasidium namibiae CBS 147.97
Aureobasidium melanogenum CBS 110374
Aureobasidium subglaciale EXF-2481
Delphinella strobiligena
Polychaeton citri CBS 116435
Dissoconium aciculare CBS 342.82
Zymoseptoria tritici IPO323
Zymoseptoria pseudotritici STIR04_2.2.1
Zymoseptoria ardabiliae STIR04_1.1.1
Dothistroma septosporum NZE10
Passalora fulva
Zasmidium cellare ATCC 36951
Pseudocercospora fijiensis
Sphaerulina musiva SO2202
Sphaerulina populicola
Cercospora zeae-maydis SCOH1-5
Hortaea acidophila
Baudoinia panamericana UAMH 10762
Teratosphaeria nubilosa
Piedraia hortae
Acidomyces richmondensis BFW
Eremomyces bilateralis CBS 781.70
Microthyrium microscopicum
Trichodelitschia bisporula
Tothia fuscella
Verruconis gallopava
Venturia inaequalis
Venturia pyrina
Aulographum hederae
Rhizodiscina lignyota
Lineolata rhizophorae
Patellaria atrata CBS 101060
Coniosporium apollinis CBS 100218
Aplosporella prunicola CBS 121167
Phyllosticta citriasiana
Neofusicoccum parvum UCRNP2
Macrophomina phaseolina MS6
Botryosphaeria dothidea
Diplodia seriata
Saccharata proteae CBS 121410
Pseudovirgaria hyperparasitica
Lepidopterella palustris CBS 459.81
Glonium stellatum
Cenococcum geophilum 1.58
Lophium mytilinum
Mytilinidion resinicola
Rhytidhysteron rufulum
Hysterium pulicare
Delitschia confertaspora
Zopfia rhizophila CBS 207.26
Lindgomyces ingoldianus
Clohesyomyces aquaticus
Amniculicola lignicola CBS 123094
Lophiotrema nucula
Polyplosphaeria fusca
Didymosphaeria enalia
Aaosphaeria arxii
Lophiostoma macrostomum CBS 122681
Westerdykella ornata
Sporormia fimetaria CBS 119925
Massariosphaeria phaeospora
Trematosphaeria pertusa
Bimuria novae-zelandiae
Paraphaeosphaeria sporulosa
Karstenula rhodostoma CBS 690.94
Massarina eburnea CBS 473.64
Byssothecium circinans
Periconia macrospinosa
Lentithecium fluviatile CBS 122367
Stagonospora sp. SRC1lsM3a
Ampelomyces quisqualis
Parastagonospora nodorum SN15
Ophiobolus disseminans
Setomelanomma holmii
Leptosphaeria maculans JN3
Plenodomus tracheiphilus IPT5
Clathrospora elynae
Decorospora gaudefroyi
Alternaria brassicicola
Alternaria alternata
Pyrenophora tritici-repentis
Pyrenophora teres f. teres 0-1
Setosphaeria turcica Et28A
Curvularia lunata m118
Bipolaris maydis C5
Bipolaris sorokiniana ND90Pr
Bipolaris oryzae ATCC 44560
Bipolaris zeicola 26-R-13
Bipolaris victoriae FI3
Cucurbitaria berberidis CBS 394.84
Pyrenochaeta sp. DS3sAY3a
Lizonia empirigonia
Didymella exigua CBS 183.55
Macroventuria anomochaeta
Dothidotthia symphoricarpi CBS 119687
Pleomassaria siparia CBS 279.74
Melanomma pulvis-pyrius CBS 109.77
Aspergillus nidulans
Coccidioides immitis
Fusarium graminearum
Neurospora crassa
Botrytis cinerea
Sclerotinia sclerotiorum
Pyronema omphalodes
Tuber melanosporum
0.10
Myriangiales
Dothideales
Capnodiales
Venturiales
Botryo-
sphaeriales
Mytilinidales
Hysteriales
Pleosporales
0
20
40
60
Number of refined clusters with a signature gene

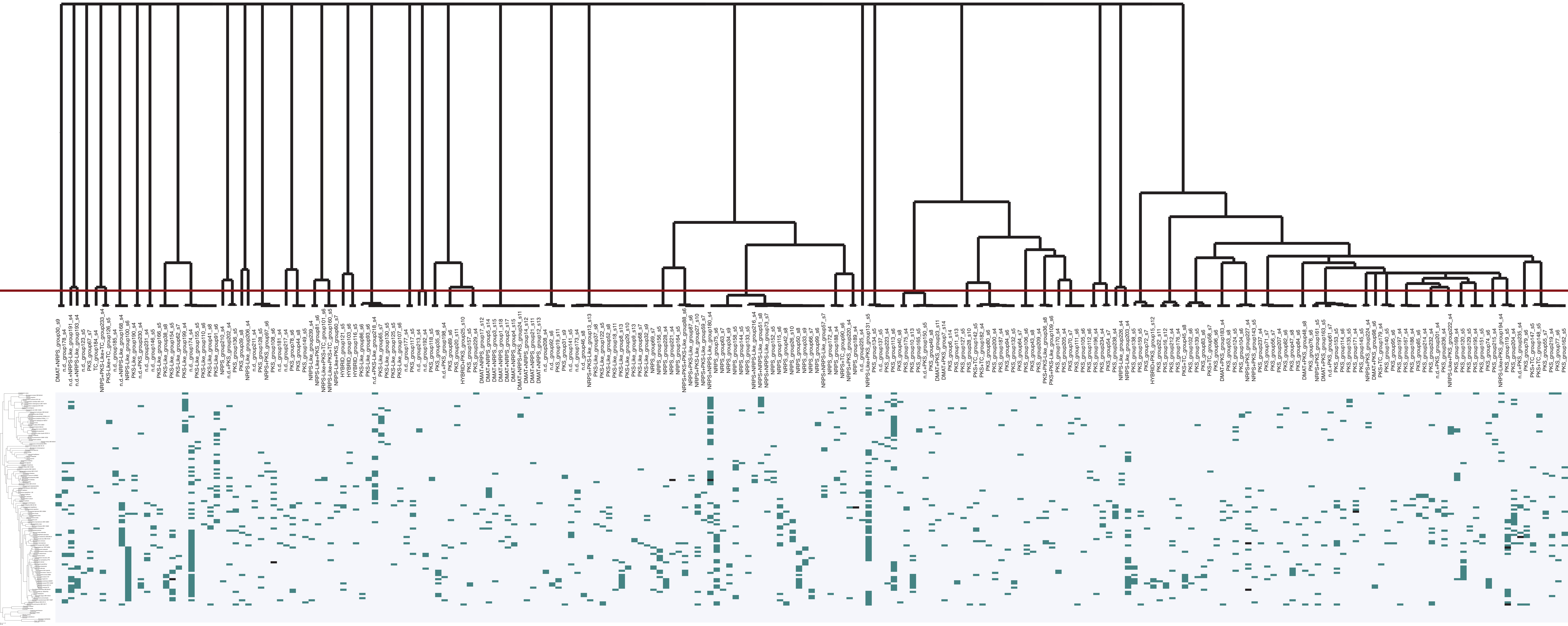

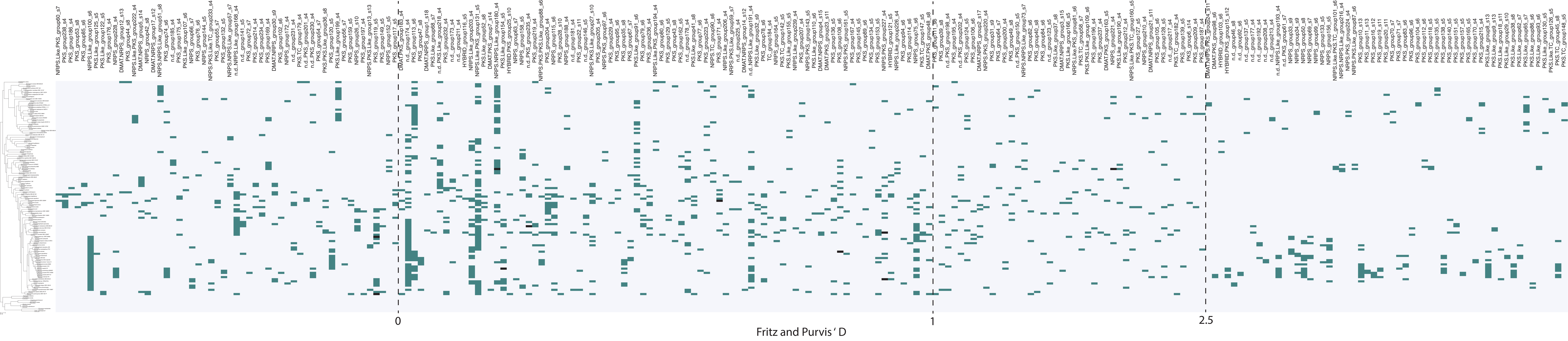
0
1
2.5
Fritz and Purvis ' D

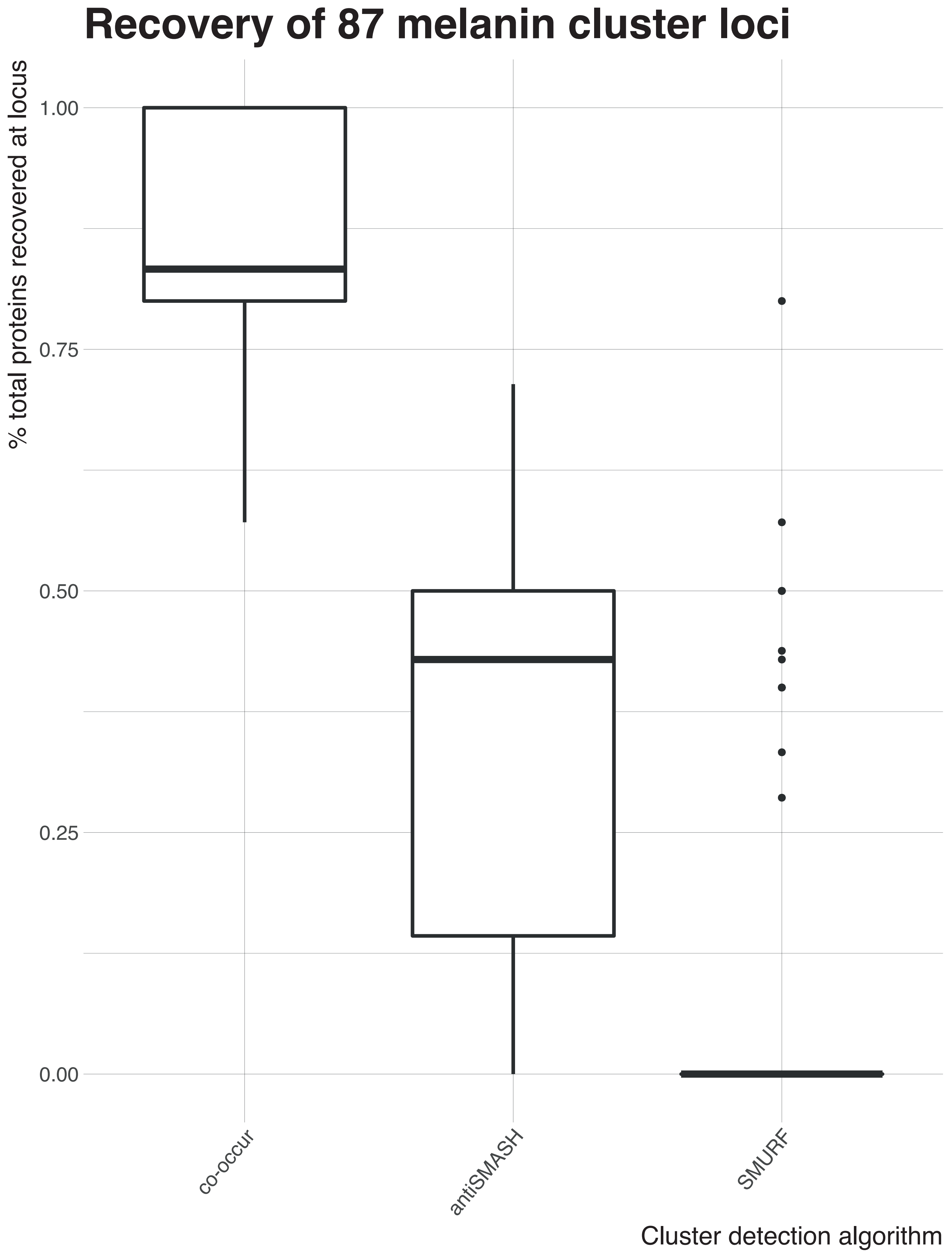
Recovery of 87 melanin cluster loci
% total proteins recovered at locus
1.00
0.75
0.50
0.25
0.00
co-occur
antiSMASH
SMURF
Cluster detection algorithm

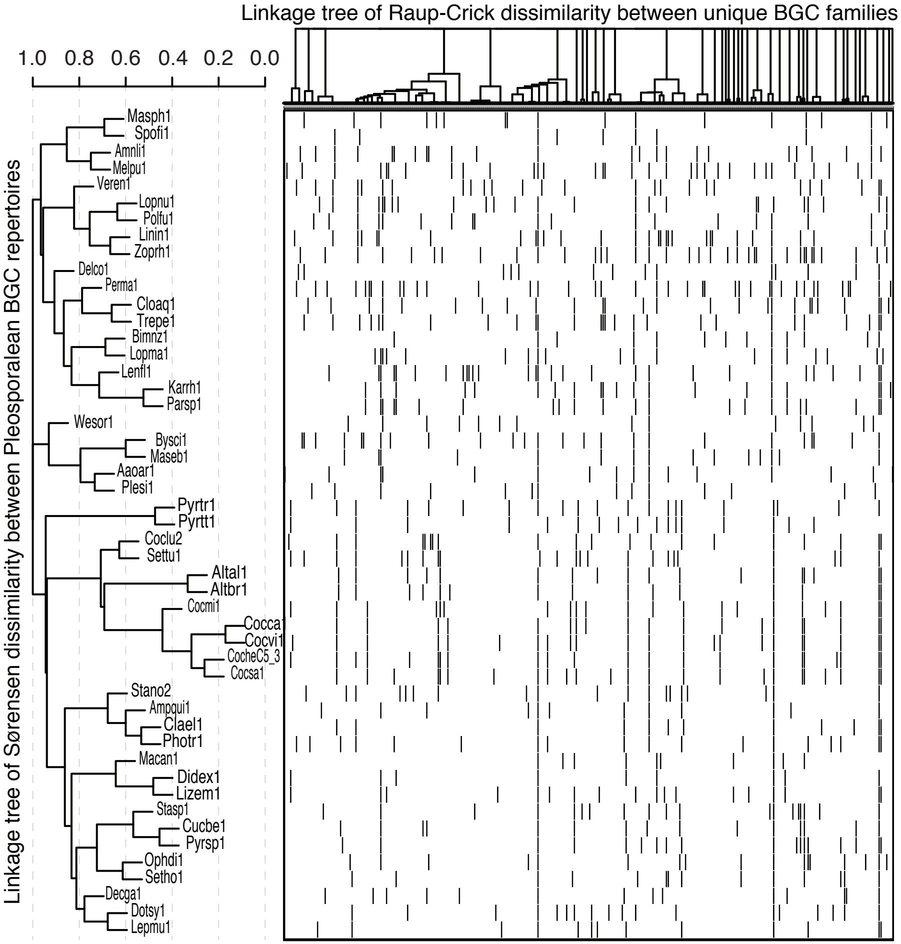

Linkage tree of Raup-Crick dissimilarity between unique BGC families
1.0 0.8 0.6 0.4 0.2 0.0
Linkage tree of Sørensen dissimilarity between Pleosporalean BGC repertoires
Masph1
Spofi1
Amnli1
Melpu1
Veren1
Lopnu1
Polfu1
Linin1
Zoprh1
Delco1
Perma1
Cloaq1
Trepe1
Bimnz1
Lopma1
Lenfl1
Karrh1
Parsp1
Wesor1
Bysci1
Maseb1
Aaoar1
Plesi1
Pyrtr1
Pyrtt1
Coclu2
Settu1
Altal1
Altbr1
Cocmi1
Cocca1
Cocvi1
CocheC5_3
Cocsa1
Stano2
Ampqui1
Clael1
Photr1
Macan1
Didex1
Lizem1
Stasp1
Cucbe1
Pyrsp1
Ophdi1
Setho1
Decga1
Dotsy1
Lepmu1

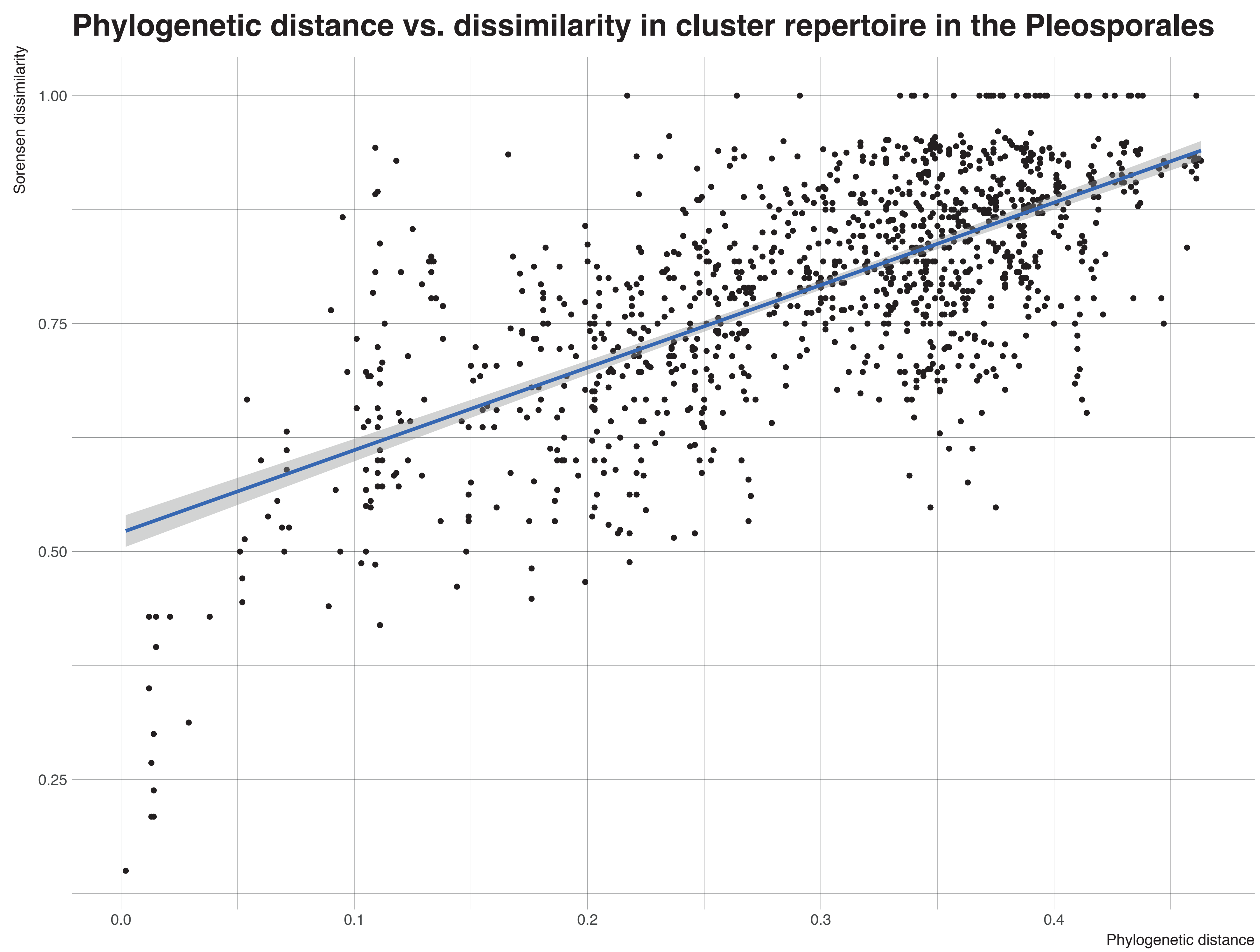

Phylogenetic distance vs. dissimilarity in cluster repertoire in the Pleosporales
Sorensen dissimilarity
1.00
0.75
0.50
0.25
0.0
0.1
0.2
0.3
0.4
Phylogenetic distance

Partitioning dissimilarity between cluster repertoires (Pleosporales)

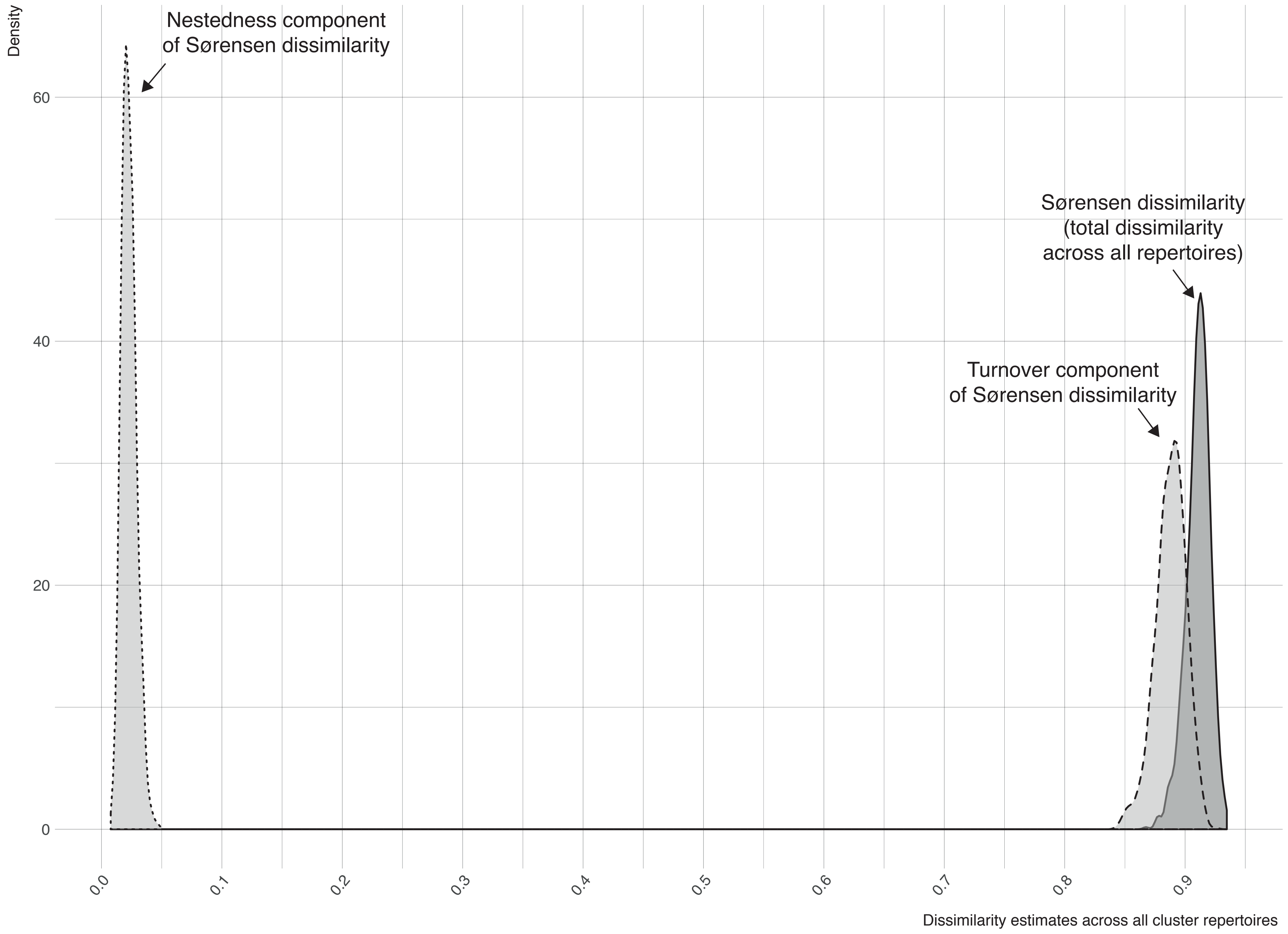

Supplement: msaa122_supplementary_data [file msaa122_supplementary_data.zip › Combined_Supplemental_Figures.pdf]
